# Photoreceptor sensory cilia and ciliopathies: focus on CEP290, RPGR and their interacting proteins

**DOI:** 10.1186/2046-2530-1-22

**Published:** 2012-12-03

**Authors:** Rivka A Rachel, Tiansen Li, Anand Swaroop

**Affiliations:** 1 Neurobiology-Neurodegeneration & Repair Laboratory, National Eye Institute, National Institutes of Health, Bethesda, MD, 20892, USA

**Keywords:** Ciliopathy, Retinal degeneration, Primary cilium, Sensory cilia, CEP290, RPGR, Bardet–Biedl syndrome, Leber congenital amaurosis, Joubert syndrome, Nephronophthisis

## Abstract

Ciliopathies encompass a broad array of clinical findings associated with genetic defects in biogenesis and/or function of the primary cilium, a ubiquitous organelle involved in the transduction of diverse biological signals. Degeneration or dysfunction of retinal photoreceptors is frequently observed in diverse ciliopathies. The sensory cilium in a photoreceptor elaborates into unique outer segment discs that provide extensive surface area for maximal photon capture and efficient visual transduction. The daily renewal of approximately 10% of outer segments requires a precise control of ciliary transport. Here, we review the ciliopathies with associated retinal degeneration, describe the distinctive structure of the photoreceptor cilium, and discuss mouse models that allow investigations into molecular mechanisms of cilia biogenesis and defects. We have specifically focused on two ciliary proteins – CEP290 and RPGR – that underlie photoreceptor degeneration and syndromic ciliopathies. Mouse models of CEP290 and RPGR disease, and of their multiple interacting partners, have helped unravel new functional insights into cell type-specific phenotypic defects in distinct ciliary proteins. Elucidation of multifaceted ciliary functions and associated protein complexes will require concerted efforts to assimilate diverse datasets from *in vivo* and *in vitro* studies. We therefore discuss a possible framework for investigating genetic networks associated with photoreceptor cilia biogenesis and pathology.

## Introduction

As the field of cilia biology has exploded over the past decade, our understanding has evolved from the initial realization of cilia as important cellular structures to the knowledge that defects in these organelles constitute a unifying framework in numerous syndromic diseases, collectively called ciliopathies. More recently, distinct sets of genes have been identified as causing overlapping symptom clusters, making it possible to link specific genetic mutations to clinical diagnosis. Amidst this rapid progress, confusion arose because disease conditions manifest as a continuum of disorders with varying severity and organ involvement rather than cleanly segregated entities. As a result, even identical gene mutations can give rise to distinct clinical manifestations, while a well-defined clinical syndrome can trace its etiologic origin to a multitude of gene defects. The goal of this review is to focus on the differences among ciliopathies based on molecular and genetic characteristics and on the realization that assigning a specific clinical diagnosis is only the starting point for identifying the culprit gene. In reaching a clear understanding of molecular mechanisms and future therapeutic strategies, correlating specific symptoms to genetic mutation(s) should provide valuable insights.

We have focused on ciliopathies that include retinal degeneration as part of the clinical spectrum in order to provide a comprehensive analysis of their mutations, phenotypes, subcellular localization of the gene products, and functional insights from respective mouse models. In addition to summarizing the current state of knowledge, we have attempted to define gaps in our understanding of cilia biology and suggested approaches for future investigations.

### An overview of ciliogenesis and cilia function

Cilia can be categorized as primary, sensory or motile. Nearly all cells develop a primary cilium, which serves either as a precursor to a cluster of motile cilia (in cells such as ventricular ependyma and tracheal epithelium) [1,2] or remains as an environmental sensor. Given that most primary cilia are now known to enable cells to interact with and respond to their environment [3], the distinction between primary and sensory cilia has lost much of its meaning. These cilia are highly specialized organelles that have developed to mediate perception of light, sound, odorants, osmolarity, pressure, flow, circulating hormones, and position within the plane of a tissue (via gradients of morphogens); these perceptions are then transmitted into the cell via signaling pathways to mediate distinct responses. For example, photoreceptor outer segments are filled with stacks of membranous discs densely packed with rhodopsin, the receptor molecule that initiates a transduction cascade turning photons into electrical signals. In the cochlea, the kinocilium serves as a transient anchor point for positioning the stereocilia bundles. In olfactory epithelium, the multiple cilia in each cell converge odorant receptors in the membrane and orchestrate G-protein coupled receptor signaling in response to environmental stimuli. Many recent reviews have summarized ciliogenesis and signaling pathways in cilia [4-13], motile cilia [14], mechanosensory cilia mechanics [15], cilia as stress and flow sensors [16], and clinical manifestations and diagnosis of neuronal pathology [17,18].

### Ciliopathies and associated pleiotropic phenotypes

Kartagener’s syndrome was one of the earliest descriptions of a motile cilia disorder [19]. While Bardet–Biedl syndrome (BBS) was recognized as a distinct collection of phenotypes at least 60 years ago [20] and Joubert syndrome (JBTS) as early as 1968 [21], ciliopathies have become recognized to have sensory cilia as a unifying theme only during the last decade [22,23]. Clinical entities that affect motile cilia only, such as Kartagener’s syndrome/primary ciliary dyskinesia, manifest *situs inversus*, bronchiectasis and sinus/respiratory complications, but lack other clinical features commonly seen in ciliopathies. The etiology of primary ciliary dyskinesia lies in genetic defects that inactivate selected molecular motors or structures within the cilia critical for motility, usually in dyneins or radial spoke components [24], thereby explaining more uniform and limited manifestations. Interestingly, defects in the sensory ciliopathies encompass a broader spectrum of gene functions including cilia biogenesis and structure, receptor trafficking and signaling, implying that sensory roles of cilia are more complex and critical to life.

**Figure 1:**
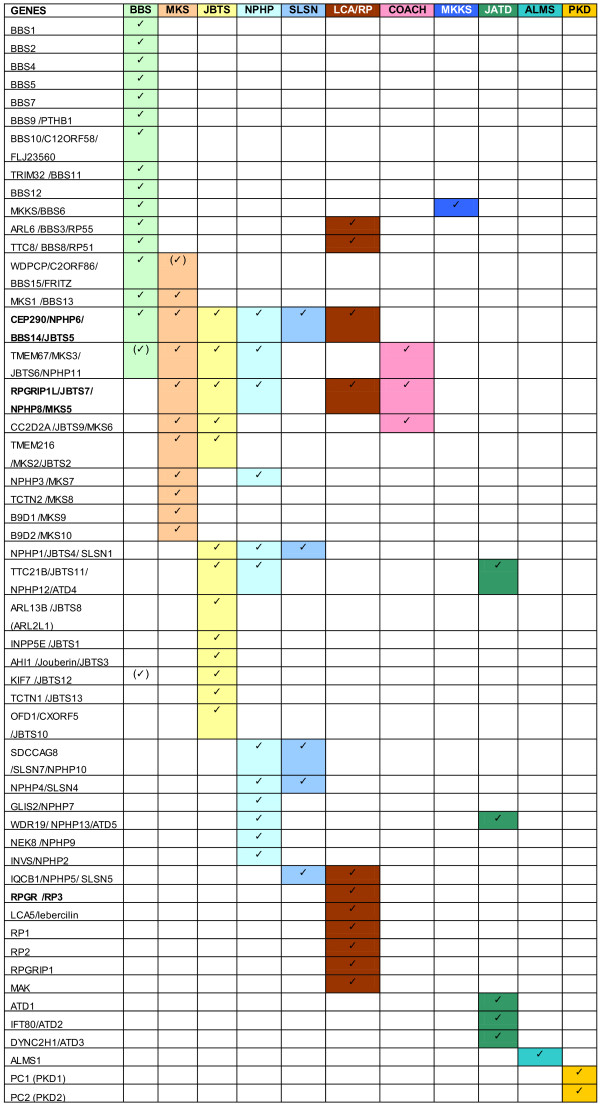
**Ciliopathy genes with syndromic manifestations.** Information from Online Mendelian Inheritance in Man (http://www.ncbi.nlm.nih.gov/omim). ALMS, Alstrom syndrome; BBS, Bardet–Biedl syndrome; COACH, Joubert syndrome with congenital hepatic fibrosis; JATD, Jeune asphyxiating thoracic dystrophy; JBTS, Joubert syndrome; LCA/RP, Leber congenital amaurosis/retinitis pigmentosa; MKKS, McKusick-Kaufman syndrome; MKS, Meckel–Gruber syndrome; NPHP, nephronophthisis; PKD, polycystic kidney disease; SLSN, Senior–Lø1ken syndrome.

Ciliopathies share an overlapping conglomeration of features, exhibiting retinal degeneration, cognitive impairments, cerebellar dysmorphogenesis, kidney cysts, hepatic fibrosis, polydactyly, situs inversus, obesity, skeletal/thoracic dysmorphology, genitourinary defects, cardiorespiratory abnormalities, neural tube patterning defects, and/or hydrocephalus (Figure 1) [25-27]. Although various syndromes may have unique symptom clusters, the distinction among clinical entities is often blurred. Clinical diagnosis alone thus provides little insight into disease etiology. Adding molecular diagnosis to classical clinical findings can be valuable in clarifying possible pathogenic mechanism(s). For example, the distinction between nephronophthisis (NPHP) and Senior–Løken syndrome (SLSN) depends on the presence of retinal findings in SLSN; however, individuals in NPHP pedigrees can also manifest ocular defects [28]. Similarly, the distinction between COACH syndrome (Joubert syndrome with congenital hepatic fibrosis) and JBTS is blurry. With a goal to establish a link between clinical features, syndromes, and genetic causes, we have summarized relevant details of many ciliopathies and their causative genes based on information from the Online Mendelian Inheritance in Man database in Figure 1. 

In contrast to motile cilia, sensory cilia are uniquely modified to carry out a particular function in a specific organ. As each tissue is designed to mediate a different sensory function, the associated pathways are more complex and slow to unravel (see Table 1). To comprehend the significance of cilia involvement, it would be helpful to delineate the pathological process in each tissue; for example, kidneys may have either massive polycystic disease or glomerulonephritis, differing pathologies that probably represent distinct etiologies. Cellular pathology and dysfunction in sensory ciliopathies may result from absent, shortened or otherwise morphologically abnormal cilia; from normal cilia structure but no transport/function; or from different cell types/tissues affected in various conditions. In photoreceptors, for example, mutations in various ciliopathy genes result in a number of distinct phenotypes, ranging from complete failure of connecting cilium formation [29] and curtailed outer segment biogenesis [30], to abnormalities of disc assembly [31]. All of these defects should eventually be traceable to a stage in ciliogenesis, transport or maintenance. Photoreceptors thus offer a unique opportunity to evaluate the contribution of ciliary proteins. 

**Table 1:** Molecular pathways associated with ciliary pathology in each affected tissue

**Organ/tissue/cell type**	**Signaling/biogenesis pathway(s)** **Wnt, Shh, PDGF, PCP**	**Reviews and other references**
Retina – photoreceptors	Ciliogenesis and transport	[32,33][34]
Cognition – brain	GPCR trafficking to neuron cilia	[35,36] JBTS: [37][38,39]
Cerebellum – granule cells?	IFT, Wnt, Shh	[40-42]
Kidney cysts	Wnt/PCP, Shh, mTOR, Ca^2+^; mechanosensation, fluid pressure, proliferation	[43-50]
Hepatic fibrosis^a^	Ductal plate malformation – PCP?; receptors expressed on cilia; cysts – hyperproliferation	[51-57][46]
Polydactyly	Shh	[58]
Situs inversus	Nodal, PCP	[59]
Obesity	Neuronal cilia receptors Shh	[60][46]
Skeletal/thoracic	Mechanical sensation, Shh, IFT	[58,61-66]
Genitourinary	Ca^2+^	[67]
Cardiorespiratory	Heart – Shh, cardiogenesis; lung – primary cilia precede motile cilia	[1,68][69][70][71]
Neural tube defects/hydrocephalus	Shh, PCP	[72][73,74]

GPCR, G-protein coupled receptor; IFT, intraflagellar transport; JBTS, Joubert syndrome; PCP, planar cell polarity; PDGF, platelet-derived growth factor. ^a^Note the importance of distinguishing primary (for example, PCP/ductal plate malformation) and secondary (for example, hepatic fibrosis and congestion) characteristics.

### Photoreceptor structure, modified cilium and transport

**Figure 2:**
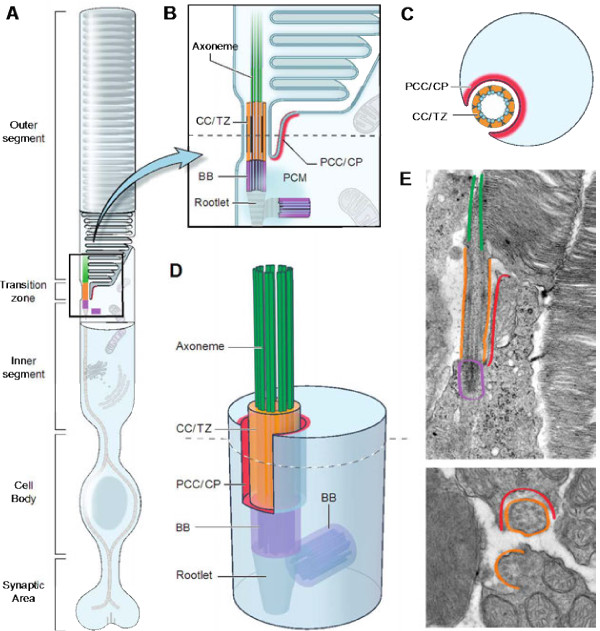
**Four distinct compartments in photoreceptor primary cilia, indicating known proteins that define their respective extent.** The four compartments are: (1) distal cilium or axoneme (Axo; green); (2) connecting cilium/transition zone (CC/TZ; orange); (3) basal body (BB; purple); and (4) periciliary complex or ciliary pocket (PCC/CP; red). These compartments serve discrete functions in the cilium. (**A**) Schematic of a photoreceptor, showing specialized domains of the cell. The primary cilium elaborates into stacks of outer segment disks packed with rhodopsin, which serves as the primary light sensors of the cell. (**B**) Enlargement of the photoreceptor transition zone in two dimensions showing the four structural and functional domains in which most ciliary proteins are expressed. These domains are identified by known protein markers, such as acetylated α-tubulin (Axo + CC/TZ) and γ-tubulin (BB). Note: illustration of outer segment is based on a traditional model of disc morphogenesis in which nascent discs are open to the extracellular milieu, but a newer model posits that new discs form within the enclosure of outer segment plasma membrane [86]. (**C**) Cross-section through the CC/TZ of the photoreceptor showing the relationship between the microtubules of the cilium and the inner segment, via the PCC/CP. (**D**) Three-dimensional representation of the transition zone and adjacent domains shown in (B). Note the manner in which the PCC surrounds the TZ. Note also that the TZ is the one compartment that contacts all other compartments. (**E**) Electron micrographs showing longitudinal (top) and cross-section (bottom) views of mouse photoreceptors. Functional domains are highlighted with the corresponding colors shown in the other panels.

The dense stacks of rhodopsin-laden discs in photoreceptor outer segments represent a highly complex and unique example of sensory cilia specialization, enlarged to house the machinery of phototransduction. As the vast majority of photoreceptors in mouse and human retina are rods, our remarks are directed primarily towards rod photoreceptors. Four ciliary compartments can be defined in photoreceptors, based on expression and other studies (Figure 2 and legend); these include the distal cilium (operationally defined as the domain occupied by Rp1 and Mak), the proximal cilium or transition zone (known in photoreceptors as the connecting cilium), the basal body, and the periciliary ridge complex or periciliary membrane complex [75,76], which is analogous to what is referred to as the ciliary pocket in general cilia literature [77]. Expression of ciliopathy-associated proteins is generally restricted to one of these four domains (Figure 2). Thus, it is now possible to divide ciliopathy proteins by discrete anatomical localization and to contemplate understanding molecular mechanisms based on precise expression data from confocal images. These proteins are identified as being expressed in specific compartments. Axo: INV/NPHP2 [43], NPHP3 [43], NPHP9/NEK8 [43], RP1, SDCCAG8 [78], MAK [79]; TZ/CC: NPHP1 [43], NPHP4 [43], NPHP8/RPGRIP1L [43], NPHP5/IQCB1 [43], NPHP6/CEP290 [43], RPGR RPGRIP1 [80-82], AHI1 [83], RP2 [84], Lebercilin [85], IFT88 [85]; BB: BBS1, BBS2, BBS3, BBS4, BBS5, BBS7, BBS9, MKKS/BBS6 [31]; PC/PCC: USH2A/usherin [75], DFNB31/USH2D/whirlin, [75], VLGR1 [75]. Having immuno-electron micrograph images of protein expression in relationship to microtubule bundles, basal body, and transition zone will further advance our understanding of protein function. 

Compartment 1, the distal cilium or axoneme, contains proteins that primarily modulate cilium length; these include MAK [79], RP1 [87], RP1L1 and IFT20 [88]. In photoreceptors, compartment 1 delineates the base of the outer segment (Figure 2B,E). Compartment 2 is referred to as the connecting cilium in photoreceptors and is equivalent to the transition zone of motile and primary cilia. Proteins in this zone include CEP290 [30,89-91], RPGR [30,92-99], RPGRIP1 [80], RPGRIP1L [100-105], IFT88 [106-108], KIF3A [109], KIF7 [110], and LCA5/Lebercilin [85]. Although intraflagellar transport proteins mediate ciliary transport along the length of the cilium, antibody localization via immunohistochemistry identifies them in specific compartments. We believe that the appearance of being concentrated in a particular subzone may reflect a bottleneck in transit. Compartment 3 comprises the basal bodies and the pericentriolar material. The proteins in this domain include BBS1 [111], BBS4 [111], BBS3 [112], MKKS [113], TTC8/BBS8 [114], and RAB8A [115]. In addition to these three core compartments, a peripheral component contributing to ciliopathies is the periciliary ridge (Compartment 4). The analogous structure in non-photoreceptor cells is the ciliary pocket [77]. The periciliary ridge was originally described in frog photoreceptors by scanning electron microscopy [76]. The same structure is not visible in mammalian photoreceptors; however, three USH2 proteins (usherin, whirlin and VLGR1) mark a functionally equivalent region, referred to as the periciliary membrane complex, to indicate a highly specialized membrane microdomain [75,116]. Distal to the basal body are structures called rootlets, which provide support for the basal bodies and cilia. A notable protein in this compartment is CROCC (rootletin) [117]. While several not strictly ciliary proteins have been included in the list of ciliopathy proteins (Figure 1) as mutations in these cause cilia-related phenotypes, a number of cilia proteins are not included – for example, trafficked cargos that are integral for outer segment function or cytoskeletal proteins that are general features of all ciliated cells.

**Figure 3:**
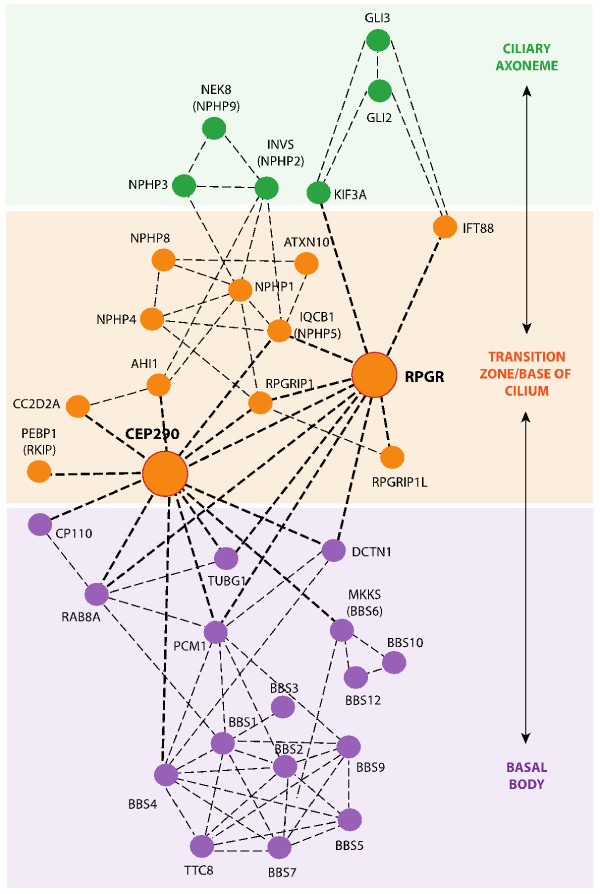
**Interactome of ciliary proteins directly or indirectly connected to CEP290 and RPGR.** Ciliary proteins directly (bold dotted lines) or indirectly (thin dotted lines) connected to CEP290 and RPGR. Ciliary expression domains are colored as in Figure 2. This network shows representative interactions.

Knowing the compartmental localization of individual proteins within the cilium will lead to new insights into cilia biogenesis and function. For example, groups of cilia proteins expressed in the same compartment, such as CEP290 and RPGR, may function in related pathways (Figure 3). Other documented interactions between proteins expressed in adjacent compartments (CEP290 and MKKS [31]; NPHP1 and NPHP4 [43]; RPGR and USH2 [118]) might provide clues to how proteins in different compartments cooperate in mediating transport or signaling. Interesting and non-exclusive possibilities are: the expression patterns indicate pools of protein accumulation rather than absolute boundaries; a system of protein relays transports cargo or signaling from the cell body to the cilia and back; interacting proteins are part of larger multi-protein complexes with overlapping boundaries; and/or discrete molecules of a given protein (for example, CEP290) form complexes within its primary compartment (transition zone), while other CEP290 domains interact with separate complexes in other compartments (for example, basal body). Given the predicted three-dimensional structure of CEP290 as a long, fibrillar coiled-coil protein, and the plethora of its interactors (Figure 3), a central role in transport and communication is suspected. 

### Retinal degeneration in clinical ciliopathies

Loss or dysfunction of photoreceptors is a moderately penetrant phenotype in ciliopathies. In the clinic, the retinal defect is called retinitis pigmentosa (RP), and in cases with an early childhood onset clinicians frequently give the diagnosis of Leber congenital amaurosis (LCA). Differences among ciliopathy phenotypes reflect both the causative gene and specific mutations within each gene. Retinal degeneration in BBS [25,119] tends to be slower compared with other ciliopathies. Patients with Meckel–Gruber syndrome have severe neural tube closure defects and early lethality; vision is thus not generally assessed in these patients [120,121]. Many JBTS patients develop a degree of childhood vision impairment [101,110,120]. Alstrom syndrome patients develop vision loss in young adulthood [122]. Like JBTS cases, patients with SLSN tend to exhibit early vision loss [34,123-125]. Visual impairment in a child can be the presenting feature of Jeune syndrome (Jeune asphyxiating thoracic dystrophy) [126].

### Mouse models of human ciliopathies with retinal degeneration

Integration of biochemical, cell biologic, protein interactions and human genetic/clinical datasets with genetic mutations in mice provides deeper insight into each gene function as it relates to cilia and pathogenic processes. Animal studies can also explain the pleiotropic nature of ciliopathies and apparent variability in clinical and disease manifestations; these may include spatial/temporal expression pattern differences, functional redundancies and variations in genetic background. Moreover, cell-based and gene-based therapies can be evaluated for toxicity and therapeutic value before moving to larger animal (dog, primate) and human studies. A large number of ciliopathy mouse models with retinal degeneration have been reported and are summarized in Table 2. Here, we focus on two ciliopathy genes involved in retinal dystrophy: CEP290, mutations in which cause up to 15 to 25% of LCA [127]; and RPGR, the most common cause of X-linked RP and one of the most frequent causes of all forms of RP [128,129]. 

**Table 2:** **Mouse models of ciliopathies with retinal degeneration**^**a**^

**Gene symbol**	**References for mouse model**	**Retinal phenotype**	**Interactors**^b^	**PR domain expressed**
**MAK**	[79]	60% ONL left at 1 month, 30% at 6 months		Axoneme
**KIF3A**	[130,131]	Intermediate rate of degeneration; 20% of wild-type ONL thickness by 10 to 12 weeks	DISC1, MAP3K11, PLEKHA5, USP7, PPP1R15A, **RPGR**	Axoneme
**RP1**	[132-136]	Slow retinal degeneration; ~40% left at 6 months	APC, MAPRE2, MAPRE3, NIF3L1, POLE	Axoneme
**CEP290**	[30,89,137]	Rapid retinal degeneration; ciliogenesis defects depending on strain	**RPGR**, **IFT88**, PCM1, DCTN1,** BBS4**, MAPK10, GNG13	CC/TZ
**AHI1/Jouberin**	[83,138,139]	Rapid – starting to go by P12; only 2 to 3 ONL rows by P24. Very few if any OS/IS	SMYD2	CC/TZ
**TMEM67/MKS3**	[140]	Early and rapid retinal degeneration	**MKS1**	CC/TZ
**IFT88/TTC10** Tg737	[106,141]	Similar to *Cep290*^*rd16*^ – failure of outer segments to elongate	**RPGR**, PRRC2A, SMNDC1, PAN3, SLC9A8	CC/TZ
**KIF7**	[110,142]	Retina not examined; mice die perinatally	USP22	CC/TZ
**LCA5** lebercilin	[85]	Rapid degeneration; between P12 and P28, reduced to 2 to 3 ONL rows. CC develops but little if any OS material.	GRIN2B, OFD1/JBTS10, IFTs	CC/TZ
**RP2**	[84,143-145]	Only information on function of the protein in transport within cells	UNC119, ARL3, YWHAB, APLP2	CC/TZ
**RPGRIP1**	[146,147]	Only three rows of ONL nuclei by 3 months of age. Overproduction of outer segments	**RPGR**, NPHP4, TFE3, SRPX, CEBPA	CC/TZ
**TCTN1**	[148]	Retina not examined; mice die prenatally	MKS1, TMEM216, TMEM67, **CEP290**, B9D1, TCTN2 AND CC2D2A.	CC/TZ
**RPGR**	[93,95,149,150]	Slow retinal degeneration	**CEP290**, **RPGRIP1**, **IFT88**, **KIF3A**, **RAB8A**	CC/TZ, BB
**ALMS1**	[151-154]	Slow degeneration – slight reduction in ONL thickness at 24 weeks; loss of OS over time; still some left at 24 weeks by rhodopsin staining	MEGF1, OFD1, TUBGCP2, TUBGCP3, TUBGCP4, **CEP290**(MS)	BB
**ARL6/BBS3**	[155]	Medium-slow retinal degeneration; hydrocephalus	**BBS1**, ARL6IP1, ARL6IP5, ARL6IP4, ARL6IP6	BB
**BBS1**	[87,156]	Slow degeneration (3 to 4 rows of ONL nuclei at 6 months); CC present but disrupted OS	BBS9, EEF1A1, ALDOB, ARL6/BBS3, PCM1	BB
**BBS2**	[157,158]	Slow degeneration – half ONL at 5 months; almost no ONL nuclei by 10 months. OS have typical indistinct, wavy pattern	EEF1A1, ALDOB, BBS7, BBS9	BB
**BBS4**	[156,159-161]	Intermediate rate of retinal degeneration; 2/3 of ONL remaining at 6 weeks; all PR lost by an unspecified adult age	PCM1, ALDOB, DCTN1, EEF1A1, EPAS1	BB
**TTC8/BBS8**	[162]	Slow degeneration – ONL half-thickness in the ‘adult’. OS maybe longer than wild-type	BBS9, PCM1, **BBS4**, **BBS1**, **BBS2**	BB
**MKKS**/**BBS6**	[74,163]	Medium-slow degeneration; bulging, disorganized OS	**CEP290**, PTN, STK16, TGIF1, ICA1	BB and proximal rootlet
**RAB8A**	[164]	Retinal phenotype has not been examined or published	**RPGR**, RABIF, BAG6, OCRL, RAB10, PQBP1	BB
**TRIM32/BBS11**	[165]	Retinal phenotype has not been examined or published	ATXN1, UBE2N, SFN, UBQLN4, UBE2V1	N/A

BB, basal body; CC/TZ, connecting cilium/transition zone; ONL, outer nuclear layer; OS/IS, outer segments/inner segments; P, postnatal day; PR, photoreceptors. ^a^Selection criteria for inclusion in Table 2: ciliopathy-related (even if no human disease has yet been described); and interaction with Rpgr or Cep290; and/or associated with retinal degeneration.

^b^Data in this column are taken primarily from entries in genecards.org. Genes in bold are CEP290 and/or RPGR interactors.

CEP290 mutations lead to a range of ciliopathy syndromes with variable clinical manifestations in humans [121,166-168] (Figure 1). Many patients present with full-spectrum ciliopathy yet select alleles cause only rapid photoreceptor degeneration (LCA) [89,127,169]. A hypomorphic allele of *Cep290*, *rd16*, has been described in mice [30]; *Cep290*^*rd16/rd16*^ mice show rapid degeneration of rod photoreceptors beginning around postnatal day 14 and leaving only residual cones by postnatal day 28 [89]. Slower cone loss and preservation of central pathways in *Cep290*^*rd16*^ mice provide opportunities for therapy [137]. Aside from the vision loss, the *Cep290*^*rd16/rd16*^ mice reveal defective olfactory transport of G proteins leading to anosmia [90] and deafness caused by cochlear hair cell dysfunction [31]. A possible mechanism of photoreceptor cell death in *Cep290*^*rd16*^ mice may involve abnormal accumulation of RKIP, the Raf-1 kinase inhibitor, which inhibits cilia formation [91]. Examination of the retinal phenotype of *Rkip*-knockout mice should shed further light on this question [170]. The *Cep290*^*rd16*^ allele is probably hypomorphic, given the expression of the protein with an in-frame deletion and a milder phenotype. Based on the human data, a null allele of *Cep290* is expected to have a severe, full-spectrum ciliopathy phenotype, which has recently been confirmed in mice (Rachel RA, Yamamoto EA, Dong L, Swaroop A, unpublished data).

In contrast to *CEP290*, *RPGR* mutations primarily cause retinal degeneration (with a few leading to syndromic phenotypes) [171-173]. The *RPGR* gene produces multiple alternatively spliced transcripts [174-176], all of which encode an N-terminal RCC1-like domain that is structurally similar to the RCC1 protein [177]. One major constitutive isoform spans exons 1 through 19 (RPGR^ex1-19^) and carries a C-terminal isoprenylation site [178]. The other major variant contains exons 1 to 14 and terminates with a large, alternative ORF15 exon (*RPGR*^*orf15*^) [128]. The RPGR-ORF15 isoform is expressed predominantly in photoreceptors [92] with some exceptions [179], concentrated in the connecting cilia [95], and appears to be the functionally important in the retina as all disease-causing mutations are present in this variant [129,172,180]. Conventional gene targeting that disrupted the RCC1-like domain abolished the expression of both types of variants in *Rpgr*-knockout mice [95]; however, unlike human patients, the retinal degeneration in this mutant is slow despite defective localization of opsins to photoreceptor outer segments. An abbreviated form of *Rpgr-orf15* transgene seems to reverse the disease phenotype in this line [94]. A naturally occurring mutant mouse (*rd9*) was shown to affect only the orf15 exon of the *Rpgr* gene [149], mimicking a majority of human patients. The retinal degeneration in the *rd9* mutation is also somewhat slow. Interestingly, a recently reported mouse conditional knockout (cko) mutant exhibited relatively earlier onset of retinal disease compared to *Rpgr*-ko and *rd9*[150]. The phenotypes in these mouse mutants are closer to what is expected in patients with *RPGR*^*orf15*^ mutations [150,172,180].

Mouse models have been generated for RPGR and CEP290 interactors and related ciliary proteins such as RPGRIP1, NPHP and BBS proteins (Figure 3). Retinal degeneration has been demonstrated in all of these models (Table 2). The retina in these mice reveal ultrastructural defects and provide insights into how cilia proteins contribute to ciliogenesis, the sequence of events in ciliogenesis, and functional interactions among specific proteins. Moreover, we can evaluate the effects of an individual allele on a uniform genetic background in mice. Such a feature allows the engineering of triple and quadruple mutants on a less variable genetic background to enable examination of the contribution of each allele to the phenotype [31,181]. Unexpectedly, such studies have begun to reveal surprising results. Rather than combinations of ciliopathy alleles necessarily resulting in a more severe phenotype, genetic findings reveal more complex relationships among different ciliary proteins. For example, loss of *Dync2h1*, involved in retrograde cilia trafficking, disrupts Sonic hedgehog signaling and cilia formation, yet combining this mutation with heterozygous loss of *Ift172*, an anterograde motor, results in a milder phenotype. *A priori* this would suggest that neutralizing two opposite forces restores equilibrium; however, reduction of the *Ift122* retrograde motor also represses the *Dync2h1* phenotype [181]. Another indication of complexity in the biogenesis and function of cilia comes from the interaction of Cep290 and Mkks, a *BBS* chaperonin protein. In this scenario, loss of *Mkks* ameliorates the sensory cilia defects in *Cep290*^*rd16*^ mutant mice, and *vice versa*[31]. Together these findings suggest a dynamic and delicate equilibrium among opposing forces in cilia function.

Mouse models with long-term photoreceptor survival and intermediate rates of disease progression should provide optimal opportunities for designing and evaluating therapies. Those with extremely rapid degeneration may not allow sufficient time for gene-based or cell-based therapy to show benefit (see, however, results on *Aipl1*^*ko/ko*^[29]), whereas those with very slow degeneration are impractical because of the required time and resource commitment. The *Rpgr*-cko and *Nrl*^*ko/ko*^*/Cep290*^*rd16/rd16*^ double mutants display appropriate characteristics for designing treatments of retinal disease caused by *RPGR* and *CEP290* mutations. *Nrl*^*ko/ko*^*/Cep290*^*rd16/rd16*^ mice have the *Cep290*^*rd16*^ mutation on the background of an all-cone photoreceptor retina due to the loss of *Nrl*; in this model, the cones survive longer than rapidly degenerating rods, allowing a longer window for treatment [89]. The *Rpgr*-cko model displays retinal degeneration that begins during the first months of life, allowing time for treatment options to be tested and evaluated [150].

For most ciliopathies, mouse models fail to completely recapitulate the human phenotype. For example, mouse *Rpgr* mutants exhibit a milder phenotype. At least nine genes have been identified as underlying Usher syndrome; yet most mouse mutants (except whirlin and usherin that cause type II Usher syndrome) bearing one of these mutations develop hearing deficits but not visual dysfunction [182]. In addition, many BBS mouse models do not, in general, develop polydactyly, unlike their human counterparts.

### Sources of complexity in ciliopathy classification

Confusion in the nomenclature of ciliopathies originates from pleiotropy of phenotypes and from variations introduced at multiple levels, including transcriptional/translational regulation, protein–protein interactions, and cellular function. Tissue-specific expression of different splice variants or protein isoforms and their subcellular localization contribute to this complexity (see Figure 2), as in the case of RPGR [99,183]. If a gene is required for cilia formation or function in all tissues, one would expect a full-spectrum ciliopathy or prenatal lethality. However, functional redundancy and tissue/cell type selectivity (for example, of *RPGR*^*orf15*^ transcript primarily in photore-ceptors) would result in a more restricted phenotypic spectrum that is also susceptible to modifier effects. In the sense that a complete disruption of ciliogenesis is incompatible with life, many ciliopathy genes would appear to be only partially required for ciliogenesis or function. Different alleles of the same gene (null vs. hypomorph vs. dominant negative) might exhibit varying severity because of the specific functional modules that are impacted [168,169,184]. Functional redundancy in genes causing a specific syndrome and phenotypic overlap among syndromes contribute greatly to complexity (Figure 1 and Table 2) [185]. Modifier genetic variants that do not cause disease on their own could modulate phenotypic spectrum of a disease-causing allele in a genetically diverse (outbred) population (such as humans) by combining alleles of different genes [103,138,169,186]. This phenomenon seems to occur in particular with BBS [187-190].

Basing diagnosis on a combination of molecular definition and clinical symptoms can help as it would clear up some of the confusion resulting from diagnosis based strictly on phenotypic manifestations. Some ciliopathies are caused by mutations in genes that are primarily associated with non-ciliopathy syndromes (for example, TRIM32/BBS11, NPHP3, and KIF7). These genes are specifically associated with pathology in certain organs; for example, NPHP3 is associated with renal–hepatic–pancreatic dysplasia, BBS11/TRIM32 causes limb girdle muscular dystrophy, and KIF7 causes acrocallosal syndrome – none of these is considered a ciliopathy. Additionally, different signaling pathways and mechanisms may operate in distinct tissues. Exceptions also exist to the rule of motile cilia having a 9 + 2 microtubule configuration and sensory cilia having a 9 + 0 structure [191]. Identifying these various sources and levels of complexity are essential. NPHP and SLSN both have kidney disease, but SLSN includes retinal degeneration; however, some patients with NPHP also have retinal disease. Mutations in only two proteins causing NPHP are so far known to also cause SLSN – that is, only mutations in those two proteins, SDCCAG8 and NPHP4, can cause RP/LCA symptoms in addition to isolated renal pathology (Figure 1). Examining whether these two proteins have retina-specific isoforms would be of interest.

### Perspectives and future directions

In this review we have discussed differences between human ciliopathies and their respective mouse models, focusing on CEP290, RPGR and their interactors. We have highlighted the importance of distinct compartments within cilia showing unique patterns of protein expression and their frequent interactions with proteins in the same or adjacent compartments. Given the complexity of these interactions, precise localization and function of each protein should provide valuable insights and testable hypotheses related to disease mechanisms. We believe that uniform analysis of tissue expression patterns is critical for elucidating the role for each gene in the retina and other relevant cell types. Expression of each isoform should be determined relative to a distinct ciliary compartment. At this stage, it is unclear for most cilia proteins whether a specific isoform is expressed in the same ciliary compartment in each tissue/cell type and whether similar mechanisms and signaling pathways are involved. Standard identifiers should thus be used to illustrate various ciliary components in colocalization studies. Commonly used markers are acetylated α-tubulin and RP1 for the distal cilium and more proximal structures, γ-tubulin for the basal body, Ush2 for the periciliary ridge complex, and rootletin (Crocc) for striated rootlets. Standardizing the data collected for each mutant and in every affected tissue/cell type will allow comparative functional analysis of specific genes. Documenting the histology of the retina with emphasis on the photoreceptor layer is required at distinct stages of degeneration. Electron microscopy in longitudinal sections and cross-sections of the junction between inner and outer segments would be helpful in determining the defects caused by mutations in a specific protein or isoform (see Figure 2). Moreover, a detailed expression pattern with respect to previously defined proteins of specific ciliary compartments will allow more precise localization.

As awareness has grown of the pivotal role of cilia in sensory signaling, various questions persist. Are activated signaling pathways specific for each ciliated tissue or cell type? Do similar multiprotein complexes play similar roles in various tissues? For example, does the composition of BBSome and NPHP–JBTS–MKS complexes [192,193] change in response to microenvironment or required cellular functions in cultured cells versus different tissues? What causes variability in genotype–phenotype correlations? For example, why do only some BBS gene mutations cause both BBS and isolated retinal dystrophy (Figure 1)? Why do only selected NPHP genes additionally cause retinal dystrophy? Do such mutations provide information about which domains of each protein may have tissue-specific functions? CEP290 and RPGR co-localize and both cause LCA/RP. Why then is only CEP290 associated with other syndromic ciliopathies even though RPGR is ubiquitously expressed?

Next-generation sequencing and new proteomics-based approaches are likely to have a major impact on the progress in this field. First, detailed analysis of ciliary localization for each protein in cultured cells and in specific tissues with relevant markers of distinct compartments will refine our understanding of cilia structure and function. Ultrastructural evaluation of photoreceptor basal bodies and connecting cilia and in other ciliated cells in mouse models will provide key information about the role of each protein. With the identification of clusters of interacting proteins [193,194], these interaction networks can be used to define relevant signaling cascades and final common pathways using biochemical and genomic techniques. A better elucidation of ciliary protein networks, their precise functional interactions and downstream signaling events would be relevant for designing therapeutic approaches that are applicable to multiple ciliopathies and pertinent for more than one specific mutation.

## Conclusions

Linking clinical diagnosis and nomenclature of ciliopathies with molecular identification depends on understanding how mutations in individual cilia genes contribute to distinct clinical phenotypes. This remains an important area of investigation. Using CEP290 and RPGR as examples of central proteins in the connecting cilium of the photoreceptor, we discuss the clinical phenotypes of mutations in these genes and in those of their interactors to illustrate this principle. We draw attention to the important conclusion that the cilium is comprised of four distinct compartments, each with discrete localization of proteins. By mapping the known interacting partners for CEP290 and RPGR, we find that hubs and disease networks, such as NPHP, BBS, and others, are concentrated in a single ciliary compartment, yet interact with members of other networks in adjacent compartments. A remaining mystery is to understand the significance of discrete localization of proteins (such as intraflagellar transport proteins) that are known to function across compartments, and the manner in which discrete networks (such as BBS and NPHP) interact with each other. These insights provide clues to the sources of complexity and confusion in the study of ciliopathies. We summarize by suggesting avenues of future pursuit that will clarify and expand the current knowledge in the field.

## Abbreviations

BB: Basal body; BBS: Bardet–Biedl syndrome; CEP290: Centrosomal protein 290 kDa; Cko: Conditional knockout; COACH: Joubert syndrome with congenital hepatic fibrosis; IFT: Intraflagellar transport; JBTS: Joubert syndrome; LCA: Leber congenital amaurosis; NPHP: Nephronophthisis; RPGR: Retinitis pigmentosa G-protein regulator; SLSN: Senior–Løken syndrome.

## Competing interests

The authors declare that they have no competing interests.

## Authors’ contributions

All authors wrote the paper and designed the figures. All authors read and approved the final version of the manuscript.

## References

[B1] Jain R Pan J Driscoll JA Wisner JW Huang T Gunsten SP You Y Brody SL Temporal relationship between primary and motile ciliogenesis in airway epithelial cells Am J Respir Cell Mol Biol 2010 43 731 739 10.1165/rcmb.2009-0328OC 20118219 PMC2993092

[B2] Sorokin SP Reconstructions of centriole formation and ciliogenesis in mammalian lungs J Cell Sci 1968 3 207 230 5661997 10.1242/jcs.3.2.207

[B3] Berbari NF O'Connor AK Haycraft CJ Yoder BK The primary cilium as a complex signaling center Curr Biol 2009 19 R526 R535 10.1016/j.cub.2009.05.025 19602418 PMC2814769

[B4] Taschner M Bhogaraju S Lorentzen E Architecture and function of IFT complex proteins in ciliogenesis Differentiation 2012 83 S12 S22 10.1016/j.diff.2011.11.001 22118932 PMC3977345

[B5] Wallingford JB Mitchell B Strange as it may seem: the many links between Wnt signaling, planar cell polarity, and cilia Genes Dev 2011 25 201 213 10.1101/gad.2008011 21289065 PMC3034894

[B6] Lee JE Gleeson JG A systems-biology approach to understanding the ciliopathy disorders Genome Med 2011 3 59 10.1186/gm275 21943201 PMC3239234

[B7] Ishikawa H Marshall WF Ciliogenesis: building the cell's antenna Nat Rev Mol Cell Biol 2011 12 222 234 10.1038/nrm3085 21427764

[B8] Silverman MA Leroux MR Intraflagellar transport and the generation of dynamic, structurally and functionally diverse cilia Trends Cell Biol 2009 19 306 316 10.1016/j.tcb.2009.04.002 19560357

[B9] Santos N Reiter JF Building it up and taking it down: the regulation of vertebrate ciliogenesis Dev Dyn 2008 237 1972 1981 10.1002/dvdy.21540 18435467 PMC3304540

[B10] Pedersen LB Rosenbaum JL Intraflagellar transport (IFT) role in ciliary assembly, resorption and signalling Curr Top Dev Biol 2008 85 23 61 19147001 10.1016/S0070-2153(08)00802-8

[B11] Gerdes JM Katsanis N Ciliary function and Wnt signal modulation Curr Top Dev Biol 2008 85 175 195 19147006 10.1016/S0070-2153(08)00807-7

[B12] Eggenschwiler JT Anderson KV Cilia and developmental signaling Annu Rev Cell Dev Biol 2007 23 345 373 10.1146/annurev.cellbio.23.090506.123249 17506691 PMC2094042

[B13] Dawe HR Farr H Gull K Centriole/basal body morphogenesis and migration during ciliogenesis in animal cells J Cell Sci 2007 120 Pt 1 7 15 17182899 10.1242/jcs.03305

[B14] Roy S The motile cilium in development and disease: emerging new insights BioEssays 2009 31 7 694 699 10.1002/bies.200900031 19492356

[B15] Hoey DA Downs ME Jacobs CR The mechanics of the primary cilium: an intricate structure with complex function J Biomech 2012 45 17 26 10.1016/j.jbiomech.2011.08.008 21899847 PMC3242821

[B16] Van der Heiden K Egorova AD Poelmann RE Wentzel JJ Hierck BP Role for primary cilia as flow detectors in the cardiovascular system Int Rev Cell Mol Biol 2011 290 87 119 21875563 10.1016/B978-0-12-386037-8.00004-1

[B17] Sattar S Gleeson JG The ciliopathies in neuronal development: a clinical approach to investigation of Joubert syndrome and Joubert syndrome-related disorders Dev Med Child Neurol 2011 53 793 798 10.1111/j.1469-8749.2011.04021.x 21679365 PMC3984879

[B18] Louvi A Grove EA Cilia in the CNS: the quiet organelle claims center stage Neuron 2011 69 1046 1060 10.1016/j.neuron.2011.03.002 21435552 PMC3070490

[B19] Kartagener M Zur pathogenese der bronchiektasien: bronchiektasien bei situs viscerum inversus Beitr Klin Tuberk 1933 83 489 501 10.1007/BF02141468

[B20] Vague J Farnarier G G S [Laurence–Moon–Bardet–Biedl syndrome] Rev Otoneuroophtalmol 1950 22 60 63 15424572

[B21] Joubert M Eisenring JJ Andermann F Familial dysgenesis of the vermis: a syndrome of hyperventilation, abnormal eye movements and retardation Neurology 1968 18 302 303 5690407

[B22] Eley L Yates LM Goodship JA Cilia and disease Curr Opin Genet Dev 2005 15 308 314 10.1016/j.gde.2005.04.008 15917207

[B23] Badano JL Katsanis N Life without centrioles: cilia in the spotlight Cell 2006 125 1228 1230 10.1016/j.cell.2006.06.013 16814708

[B24] Afzelius BA Genetical and ultrastructural aspects of the immotile-cilia syndrome Am J Hum Genet 1981 33 852 864 7034533 PMC1685161

[B25] Beales PL Elcioglu N Woolf AS Parker D Flinter FA New criteria for improved diagnosis of Bardet–Biedl syndrome: results of a population survey J Med Genet 1999 36 437 446 10874630 PMC1734378

[B26] Waters AM Beales PL Pagon RA, Bird TD, Dolan CR, Stephens K Bardet–Biedl syndrome GeneReviews 2003 University of Washington Jul 14 [updated 2011 Sep 29]

[B27] Parisi MA Clinical and molecular features of Joubert syndrome and related disorders Am J Med Genet C Semin Med Genet 2009 151C 326 340 10.1002/ajmg.c.30229 19876931 PMC2797758

[B28] Otto EA Tory K Attanasio M Zhou W Chaki M Paruchuri Y Wise EL Wolf MT Utsch B Becker C Nurnberg G Nurnberg P Nayir A Saunier S Antignac C Hildebrandt F Hypomorphic mutations in meckelin (MKS3/TMEM67) cause nephronophthisis with liver fibrosis (NPHP11) J Med Genet 2009 46 663 670 10.1136/jmg.2009.066613 19508969

[B29] Sun X Pawlyk B Xu X Liu X Bulgakov OV Adamian M Sandberg MA Khani SC Tan MH Smith AJ Ali RR Li T Gene therapy with a promoter targeting both rods and cones rescues retinal degeneration caused by AIPL1 mutations Gene Ther 2010 17 117 131 10.1038/gt.2009.104 19710705 PMC2804971

[B30] Chang B Khanna H Hawes N Jimeno D He S Lillo C Parapuram SK Cheng H Scott A Hurd RE Sayer JA Otto EA Attanasio M O'Toole JF Jin G Shou C Hildebrandt F Williams DS Heckenlively JR Swaroop A In-frame deletion in a novel centrosomal/ciliary protein CEP290/NPHP6 perturbs its interaction with RPGR and results in early-onset retinal degeneration in the rd16 mouse Hum Mol Genet 2006 15 1847 1857 10.1093/hmg/ddl107 16632484 PMC1592550

[B31] Rachel RA May-Simera HL Veleri S Gotoh N Choi BY Murga-Zamalloa C McIntyre JC Marek J Lopez I Hackett AN Brooks M den Hollander AI Beales PL Li T Jacobson SG Sood R Martens JR Liu P Friedman TB Khanna H Koenekoop RK Kelley MW Swaroop A Combining Cep290 and Mkks ciliopathy alleles in mice rescues sensory defects and restores ciliogenesis J Clin Investig 2012 122 1233 1245 10.1172/JCI60981 22446187 PMC3314468

[B32] Mockel A Perdomo Y Stutzmann F Letsch J Marion V Dollfus H Retinal dystrophy in Bardet–Biedl syndrome and related syndromic ciliopathies Prog Retin Eye Res 2011 30 258 274 10.1016/j.preteyeres.2011.03.001 21477661

[B33] Ramamurthy V Cayouette M Development and disease of the photoreceptor cilium Clin Genet 2009 76 137 145 10.1111/j.1399-0004.2009.01240.x 19790290

[B34] Adams NA Awadein A Toma HS The retinal ciliopathies Ophthalmic Genet 2007 28 113 125 10.1080/13816810701537424 17896309

[B35] Domire JS Green JA Lee KG Johnson AD Askwith CC Mykytyn K Dopamine receptor 1 localizes to neuronal cilia in a dynamic process that requires the Bardet–Biedl syndrome proteins Cell Mol Life Sci 2011 68 2951 2960 10.1007/s00018-010-0603-4 21152952 PMC3368249

[B36] Green JA Mykytyn K Neuronal ciliary signaling in homeostasis and disease Cell Mol Life Sci 2010 67 3287 3297 10.1007/s00018-010-0425-4 20544253 PMC3349968

[B37] Doherty D Joubert syndrome: insights into brain development, cilium biology, and complex disease Semin Pediatr Neurol 2009 16 143 154 10.1016/j.spen.2009.06.002 19778711 PMC2804071

[B38] Whitfield JF The neuronal primary cilium – an extrasynaptic signaling device Cell Signal 2004 16 763 767 10.1016/j.cellsig.2003.12.002 15115655

[B39] Whitfield JF Chakravarthy BR The neuronal primary cilium: driver of neurogenesis and memory formation in the hippocampal dentate gyrus? Cell Signal 2009 21 1351 1355 10.1016/j.cellsig.2009.02.013 19249355

[B40] Louie CM Gleeson JG Genetic basis of Joubert syndrome and related disorders of cerebellar development Hum Mol Genet 2005 14 235 242 10.1093/hmg/ddi264 16244321

[B41] Millen KJ Gleeson JG Cerebellar development and disease Curr Opin Neurobiol 2008 18 12 19 10.1016/j.conb.2008.05.010 18513948 PMC2474776

[B42] Spassky N Han YG Aguilar A Strehl L Besse L Laclef C Ros MR Garcia-Verdugo JM Alvarez-Buylla A Primary cilia are required for cerebellar development and Shh-dependent expansion of progenitor pool Dev Biol 2008 317 246 259 10.1016/j.ydbio.2008.02.026 18353302 PMC4043448

[B43] Shiba D Yokoyama T The ciliary transitional zone and nephrocystins Differentiation 2012 83 S91 S96 Epub 2011 Dec 12 10.1016/j.diff.2011.11.006 22169048

[B44] Winyard P Jenkins D Putative roles of cilia in polycystic kidney disease Biochim Biophys Acta 2011 1812 1256 1262 10.1016/j.bbadis.2011.04.012 21586324

[B45] Takiar V Caplan MJ Polycystic kidney disease: pathogenesis and potential therapies Biochim Biophys Acta 2011 1812 1337 1343 10.1016/j.bbadis.2010.11.014 21146605 PMC3139769

[B46] D'Angelo A Franco B The primary cilium in different tissues-lessons from patients and animal models Pediatr Nephrol 2011 26 655 662 10.1007/s00467-010-1650-7 20890766

[B47] Dalagiorgou G Basdra EK Papavassiliou AG Polycystin-1: function as a mechanosensor Int J BiochemCell Biol 2010 42 1610 1613 10.1016/j.biocel.2010.06.017 20601082

[B48] Hurd TW Hildebrandt F Mechanisms of nephronophthisis and related ciliopathies Nephron Exp Nephrol 2011 118 e9 e14 10.1159/000320888 21071979 PMC2992643

[B49] Hildebrandt F Attanasio M Otto E Nephronophthisis: disease mechanisms of a ciliopathy J Am Soc Nephrol 2009 20 23 35 10.1681/ASN.2008050456 19118152 PMC2807379

[B50] Lancaster MA Louie CM Silhavy JL Sintasath L Decambre M Nigam SK Willert K Gleeson JG Impaired Wnt-beta-catenin signaling disrupts adult renal homeostasis and leads to cystic kidney ciliopathy Nat Med 2009 15 1046 1054 10.1038/nm.2010 19718039 PMC2895985

[B51] Gunay-Aygun M Liver and kidney disease in ciliopathies Am J Med Genet C Semin Med Genet 2009 151C 296 306 10.1002/ajmg.c.30225 19876928 PMC2919058

[B52] Larusso NF Masyuk TV The role of cilia in the regulation of bile flow Dig Dis 2011 29 6 12 10.1159/000324121 21691098 PMC3128136

[B53] Masyuk T Masyuk A LaRusso N Cholangiociliopathies: genetics, molecular mechanisms and potential therapies Curr Opin Gastroenterol 2009 25 265 271 10.1097/MOG.0b013e328328f4ff 19349863 PMC3831343

[B54] Masyuk AI Masyuk TV LaRusso NF Cholangiocyte primary cilia in liver health and disease Dev Dyn 2008 237 2007 2012 10.1002/dvdy.21530 18407555 PMC2574848

[B55] Masyuk AI Gradilone SA Banales JM Huang BQ Masyuk TV Lee SO Splinter PL Stroope AJ Larusso NF Cholangiocyte primary cilia are chemosensory organelles that detect biliary nucleotides via P2Y12 purinergic receptors Am J Physiol Gastrointest Liver Physiol 2008 295 G725 G734 10.1152/ajpgi.90265.2008 18687752 PMC2575915

[B56] Gradilone SA Masyuk AI Splinter PL Banales JM Huang BQ Tietz PS Masyuk TV Larusso NF Cholangiocyte cilia express TRPV4 and detect changes in luminal tonicity inducing bicarbonate secretion Proc Natl Acad Sci U S A 2007 104 19138 19143 10.1073/pnas.0705964104 18024594 PMC2141921

[B57] Masyuk AI Masyuk TV Splinter PL Huang BQ Stroope AJ LaRusso NF Cholangiocyte cilia detect changes in luminal fluid flow and transmit them into intracellular Ca2+ and cAMP signaling Gastroenterology 2006 131 911 920 10.1053/j.gastro.2006.07.003 16952559 PMC1866168

[B58] Bimonte S De Angelis A Quagliata L Giusti F Tammaro R Dallai R Ascenzi MG Diez-Roux G Franco B Ofd1 is required in limb bud patterning and endochondral bone development Dev Biol 2011 349 179 191 10.1016/j.ydbio.2010.09.020 20920500

[B59] Marion V Stutzmann F Gerard M De Melo C Schaefer E Claussmann A Helle S Delague V Souied E Barrey C Verloes A Stoetzel C Dollfus H Exome sequencing identifies mutations in LZTFL1, a BBSome and smoothened trafficking regulator, in a family with Bardet–Biedl syndrome with situs inversus and insertional polydactyly J Med Genet 2012 49 317 21 Epub 2012 Apr 17 10.1136/jmedgenet-2012-100737 22510444

[B60] Berbari NF Johnson AD Lewis JS Askwith CC Mykytyn K Identification of ciliary localization sequences within the third intracellular loop of G protein-coupled receptors Mol Biol Cell 2008 19 1540 1547 10.1091/mbc.E07-09-0942 18256283 PMC2291422

[B61] Anderson CT Castillo AB Brugmann SA Helms JA Jacobs CR Stearns T Primary cilia: cellular sensors for the skeleton Anat Rec 2008 291 1074 1078 10.1002/ar.20754 PMC2879613 18727074

[B62] Malone AM Anderson CT Stearns T Jacobs CR Primary cilia in bone J MusculoskeletNeuronal Interact 2007 7 301 18094482

[B63] Malone AM Anderson CT Tummala P Kwon RY Johnston TR Stearns T Jacobs CR Primary cilia mediate mechanosensing in bone cells by a calcium-independent mechanism Proc Natl Acad Sci U S A 2007 104 13325 13330 10.1073/pnas.0700636104 17673554 PMC1939687

[B64] Haycraft CJ Zhang Q Song B Jackson WS Detloff PJ Serra R Yoder BK Intraflagellar transport is essential for endochondral bone formation Development 2007 134 307 316 10.1242/dev.02732 17166921

[B65] Liu A Wang B Niswander LA Mouse intraflagellar transport proteins regulate both the activator and repressor functions of Gli transcription factors Development 2005 132 3103 3111 10.1242/dev.01894 15930098

[B66] Weatherbee SD Niswander LA Anderson KV A mouse model for Meckel syndrome reveals Mks1 is required for ciliogenesis and Hedgehog signaling Hum Mol Genet 2009 18 4565 4575 10.1093/hmg/ddp422 19776033 PMC2773271

[B67] Cattaneo I Condorelli L Terrinoni AR Antiga L Sangalli F Remuzzi A Shear stress reverses dome formation in confluent renal tubular cells Cell Physiol Biochem 2011 28 673 682 10.1159/000335813 22178879

[B68] Clement CA Kristensen SG Mollgard K Pazour GJ Yoder BK Larsen LA Christensen ST The primary cilium coordinates early cardiogenesis and hedgehog signaling in cardiomyocyte differentiation J Cell Sci 2009 122 Pt 17 3070 3082 19654211 10.1242/jcs.049676 PMC2729259

[B69] You Y Huang T Richer EJ Schmidt JE Zabner J Borok Z Brody SL Role of f-box factor foxj1 in differentiation of ciliated airway epithelial cells Am J Physiol Lung Cell Mol Physiol 2004 286 L650 L657 12818891 10.1152/ajplung.00170.2003

[B70] Patel AC Brody SL Stappenbeck TS Holtzman MJ Tracking cell lineage to rediscover (again) the switch from ciliated to mucous cells Amer J Respir Cell Mol Biol 2011 44 261 263 21364232 10.1165/rcmb.2010-0468ed

[B71] Brody SL Yan XH Wuerffel MK Song SK Shapiro SD Ciliogenesis and left-right axis defects in forkhead factor HFH-4-null mice Am J Respir Cell Mol Biol 2000 23 45 51 10873152 10.1165/ajrcmb.23.1.4070

[B72] Horner VL Caspary T Disrupted dorsal neural tube BMP signaling in the cilia mutant Arl13b hnn stems from abnormal Shh signaling Dev Biol 2011 355 43 54 10.1016/j.ydbio.2011.04.019 21539826 PMC3119544

[B73] Murdoch JN Copp AJ The relationship between sonic Hedgehog signaling, cilia, and neural tube defects Birth Defects Res A Clin Mol Teratol 2010 88 633 652 10.1002/bdra.20686 20544799 PMC3635124

[B74] Ross AJ May-Simera H Eichers ER Kai M Hill J Jagger DJ Leitch CC Chapple JP Munro PM Fisher S Tan PL Phillips HM Leroux MR Henderson DJ Murdoch JN Copp AJ Eliot MM Lupski JR Kemp DT Dollfus H Tada M Katsanis N Forge A Beales PL Disruption of Bardet–Biedl syndrome ciliary proteins perturbs planar cell polarity in vertebrates Nat Genet 2005 37 1135 1140 10.1038/ng1644 16170314

[B75] Yang J Liu X Zhao Y Adamian M Pawlyk B Sun X McMillan DR Liberman MC Li T Ablation of whirlin long isoform disrupts the USH2 protein complex and causes vision and hearing loss PLoS Genet 2010 6 e1000955 10.1371/journal.pgen.1000955 20502675 PMC2873905

[B76] Peters KR Palade GE Schneider BG Papermaster DS Fine structure of a periciliary ridge complex of frog retinal rod cells revealed by ultrahigh resolution scanning electron microscopy J Cell Biol 1983 96 265 276 10.1083/jcb.96.1.265 6219117 PMC2112274

[B77] Ghossoub R Molla-Herman A Bastin P Benmerah A The ciliary pocket: a once-forgotten membrane domain at the base of cilia Biol Cell 2011 103 131 144 10.1042/BC20100128 21275905

[B78] Otto EA Hurd TW Airik R Chaki M Zhou W Stoetzel C Patil SB Levy S Ghosh AK Murga-Zamalloa CA van Reeuwijk J Letteboer SJ Sang L Giles RH Liu Q Coene KL Estrada-Cuzcano A Collin RW McLaughlin HM Held S Kasanuki JM Ramaswami G Conte J Lopez I Washburn J Macdonald J Hu J Yamashita Y Maher ER Guay-Woodford LM Candidate exome capture identifies mutation of SDCCAG8 as the cause of a retinal–renal ciliopathy Nat Genet 2010 42 840 850 10.1038/ng.662 20835237 PMC2947620

[B79] Omori Y Chaya T Katoh K Kajimura N Sato S Muraoka K Ueno S Koyasu T Kondo M Furukawa T Negative regulation of ciliary length by ciliary male germ cell-associated kinase (Mak) is required for retinal photoreceptor survival Proc Natl Acad Sci U S A 2010 107 22671 22676 10.1073/pnas.1009437108 21148103 PMC3012466

[B80] Hong DH Yue G Adamian M Li T Retinitis pigmentosa GTPase regulator (RPGRr)-interacting protein is stably associated with the photoreceptor ciliary axoneme and anchors RPGR to the connecting cilium J Biol Chem 2001 276 15 12091 12099 10.1074/jbc.M009351200 11104772

[B81] Pawlyk BS Smith AJ Buch PK Adamian M Hong DH Sandberg MA Ali RR Li T Gene replacement therapy rescues photoreceptor degeneration in a murine model of Leber congenital amaurosis lacking RPGRIP Invest Ophthalmol Vis Sci 2005 46 3039 3045 10.1167/iovs.05-0371 16123399

[B82] Zhao Y Hong DH Pawlyk B Yue G Adamian M Grynberg M Godzik A Li T The retinitis pigmentosa GTPase regulator (RPGR)-interacting protein: subserving RPGR function and participating in disk morphogenesis Proc Natl Acad Sci U S A 2003 100 3965 3970 10.1073/pnas.0637349100 12651948 PMC153031

[B83] Westfall JE Hoyt C Liu Q Hsiao YC Pierce EA Page-McCaw PS Ferland RJ Retinal degeneration and failure of photoreceptor outer segment formation in mice with targeted deletion of the Joubert syndrome gene, Ahi1 J Neurosci 2010 30 8759 8768 10.1523/JNEUROSCI.5229-09.2010 20592197 PMC2923804

[B84] Holopainen JM Cheng CL Molday LL Johal G Coleman J Dyka F Hii T Ahn J Molday RS Interaction and localization of the retinitis pigmentosa protein RP2 and NSF in retinal photoreceptor cells Biochemistry 2010 49 7439 7447 10.1021/bi1005249 20669900 PMC2942077

[B85] Boldt K Mans DA Won J van Reeuwijk J Vogt A Kinkl N Letteboer SJ Hicks WL Hurd RE Naggert JK Texier Y den Hollander AI Koenekoop RK Bennett J Cremers FP Gloeckner CJ Nishina PM Roepman R Ueffing M Disruption of intraflagellar protein transport in photoreceptor cilia causes Leber congenital amaurosis in humans and mice J Clin Invest 2011 121 2169 2180 10.1172/JCI45627 21606596 PMC3104757

[B86] Chuang JZ Zhao Y Sung CH SARA-regulated vesicular targeting underlies formation of the light-sensing organelle in mammalian rods Cell 2007 130 3 535 547 10.1016/j.cell.2007.06.030 17693260 PMC3857750

[B87] Kulaga HM Leitch CC Eichers ER Badano JL Lesemann A Hoskins BE Lupski JR Beales PL Reed RR Katsanis N Loss of BBS proteins causes anosmia in humans and defects in olfactory cilia structure and function in the mouse Nat Genet 2004 36 9 994 998 10.1038/ng1418 15322545

[B88] Sedmak T Wolfrum U Intraflagellar transport molecules in ciliary and nonciliary cells of the retina J Cell Biol 2010 189 171 186 10.1083/jcb.200911095 20368623 PMC2854383

[B89] Cideciyan AV Rachel RA Aleman TS Swider M Schwartz SB Sumaroka A Roman AJ Stone EM Jacobson SG Swaroop A Cone photoreceptors are the main targets for gene therapy of NPHP5 (IQCB1) or NPHP6 (CEP290) blindness: generation of an all-cone Nphp6 hypomorph mouse that mimics the human retinal ciliopathy Hum Mol Genet 2011 20 1411 1423 10.1093/hmg/ddr022 21245082 PMC3049361

[B90] McEwen DP Koenekoop RK Khanna H Jenkins PM Lopez I Swaroop A Martens JR Hypomorphic CEP290/NPHP6 mutations result in anosmia caused by the selective loss of G proteins in cilia of olfactory sensory neurons Proc Natl Acad Sci U S A 2007 104 15917 15922 10.1073/pnas.0704140104 17898177 PMC2000398

[B91] Murga-Zamalloa CA Ghosh AK Patil SB Reed NA Chan LS Davuluri S Peranen J Hurd TW Rachel RA Khanna H Accumulation of the Raf-1 kinase inhibitory protein (Rkip) is associated with Cep290-mediated photoreceptor degeneration in ciliopathies J Biol Chem 2011 286 28276 28286 10.1074/jbc.M111.237560 21685394 PMC3151072

[B92] Hong DH Pawlyk B Sokolov M Strissel KJ Yang J Tulloch B Wright AF Arshavsky VY Li T RPGR isoforms in photoreceptor connecting cilia and the transitional zone of motile cilia Invest Ophthalmol Vis Sci 2003 44 2413 2421 10.1167/iovs.02-1206 12766038

[B93] Brunner S Skosyrski S Kirschner-Schwabe R Knobeloch KP Neidhardt J Feil S Glaus E Luhmann UF Ruther K Berger W Cone versus rod disease in a mutant Rpgr mouse caused by different genetic backgrounds Invest Ophthalmol Vis Sci 2010 51 1106 1115 10.1167/iovs.08-2742 20007830

[B94] Hong DH Pawlyk BS Adamian M Sandberg MA Li T A single, abbreviated RPGR-ORF15 variant reconstitutes RPGR function in vivo Invest Ophthalmol Vis Sci 2005 46 435 441 10.1167/iovs.04-1065 15671266

[B95] Hong DH Pawlyk BS Shang J Sandberg MA Berson EL Li T A retinitis pigmentosa GTPase regulator (RPGR)-deficient mouse model for X-linked retinitis pigmentosa (RP3) Proc Natl Acad Sci U S A 2000 97 3649 3654 10.1073/pnas.97.7.3649 10725384 PMC16294

[B96] Hosch J Lorenz B Stieger K RPGR: role in the photoreceptor cilium, human retinal disease, and gene therapy Ophthalmic Genet 2011 32 1 11 10.3109/13816810.2010.535889 21174525

[B97] Kirschner R Rosenberg T Schultz-Heienbrok R Lenzner S Feil S Roepman R Cremers FP Ropers HH Berger W RPGR transcription studies in mouse and human tissues reveal a retina-specific isoform that is disrupted in a patient with X-linked retinitis pigmentosa Hum Mol Genet 1999 8 1571 1578 10.1093/hmg/8.8.1571 10401007

[B98] Mavlyutov TA Zhao H Ferreira PA Species-specific subcellular localization of RPGR and RPGRIP isoforms: implications for the phenotypic variability of congenital retinopathies among species Hum Mol Genet 2002 11 1899 1907 10.1093/hmg/11.16.1899 12140192

[B99] Murga-Zamalloa C Swaroop A Khanna H Multiprotein complexes of Retinitis Pigmentosa GTPase regulator (RPGR), a ciliary protein mutated in X-linked Retinitis Pigmentosa (XLRP) Adv Exp Med Biol 2010 664 105 114 10.1007/978-1-4419-1399-9_13 20238008 PMC3464500

[B100] Arts HH Doherty D van Beersum SE Parisi MA Letteboer SJ Gorden NT Peters TA Marker T Voesenek K Kartono A Ozyurek H Farin FM Kroes HY Wolfrum U Brunner HG Cremers FP Glass IA Knoers NV Roepman R Mutations in the gene encoding the basal body protein RPGRIP1L, a nephrocystin-4 interactor, cause Joubert syndrome Nat Genet 2007 39 882 888 10.1038/ng2069 17558407

[B101] Brancati F Travaglini L Zablocka D Boltshauser E Accorsi P Montagna G Silhavy JL Barrano G Bertini E Emma F Rigoli L Dallapiccola B Gleeson JG Valente EM RPGRIP1L mutations are mainly associated with the cerebello-renal phenotype of Joubert syndrome-related disorders Clin Genet 2008 74 164 170 10.1111/j.1399-0004.2008.01047.x 18565097 PMC2752690

[B102] Delous M Baala L Salomon R Laclef C Vierkotten J Tory K Golzio C Lacoste T Besse L Ozilou C Moutkine I Hellman NE Anselme I Silbermann F Vesque C Gerhardt C Rattenberry E Wolf MT Gubler MC Martinovic J Encha-Razavi F Boddaert N Gonzales M Macher MA Nivet H Champion G Bertheleme JP Niaudet P McDonald F Hildebrandt F The ciliary gene RPGRIP1L is mutated in cerebello-oculo-renal syndrome (Joubert syndrome type B) and Meckel syndrome Nat Genet 2007 39 875 881 10.1038/ng2039 17558409

[B103] Khanna H Davis EE Murga-Zamalloa CA Estrada-Cuzcano A Lopez I den Hollander AI Zonneveld MN Othman MI Waseem N Chakarova CF Maubaret C Diaz-Font A MacDonald I Muzny DM Wheeler DA Morgan M Lewis LR Logan CV Tan PL Beer MA Inglehearn CF Lewis RA Jacobson SG Bergmann C Beales PL Attie-Bitach T Johnson CA Otto EA Bhattacharya SS Hildebrandt F A common allele in RPGRIP1L is a modifier of retinal degeneration in ciliopathies Nat Genet 2009 41 739 745 10.1038/ng.366 19430481 PMC2783476

[B104] Liu L Zhang M Xia Z Xu P Chen L Xu T Caenorhabditis elegans ciliary protein NPHP-8, the homologue of human RPGRIP1L, is required for ciliogenesis and chemosensation Biochem Biophys Res Commun 2011 410 626 631 10.1016/j.bbrc.2011.06.041 21689635

[B105] Wolf MT Saunier S O'Toole JF Wanner N Groshong T Attanasio M Salomon R Stallmach T Sayer JA Waldherr R Griebel M Oh J Neuhaus TJ Josefiak U Antignac C Otto EA Hildebrandt F Mutational analysis of the RPGRIP1L gene in patients with Joubert syndrome and nephronophthisis Kidney Int 2007 72 1520 1526 10.1038/sj.ki.5002630 17960139

[B106] Pazour GJ Baker SA Deane JA Cole DG Dickert BL Rosenbaum JL Witman GB Besharse JC The intraflagellar transport protein, IFT88, is essential for vertebrate photoreceptor assembly and maintenance J Cell Biol 2002 157 103 113 10.1083/jcb.200107108 11916979 PMC2173265

[B107] Sukumaran S Perkins BD Early defects in photoreceptor outer segment morphogenesis in zebrafish ift57, ift88 and ift172 intraflagellar transport mutants Vision Res 2009 49 479 489 10.1016/j.visres.2008.12.009 19136023 PMC2674962

[B108] Tsujikawa M Malicki J Intraflagellar transport genes are essential for differentiation and survival of vertebrate sensory neurons Neuron 2004 42 703 716 10.1016/S0896-6273(04)00268-5 15182712

[B109] Whitehead JL Wang SY Bost-Usinger L Hoang E Frazer KA Burnside B Photoreceptor localization of the KIF3A and KIF3B subunits of the heterotrimeric microtubule motor kinesin II in vertebrate retina Exp Eye Res 1999 69 491 503 10.1006/exer.1999.0724 10548469

[B110] Dafinger C Liebau MC Elsayed SM Hellenbroich Y Boltshauser E Korenke GC Fabretti F Janecke AR Ebermann I Nurnberg G Nurnberg P Zentgraf H Koerber F Addicks K Elsobky E Benzing T Schermer B Bolz HJ Mutations in KIF7 link Joubert syndrome with Sonic Hedgehog signaling and microtubule dynamics J Clin Invest 2011 121 2662 2667 10.1172/JCI43639 21633164 PMC3223820

[B111] Gerdes JM Liu Y Zaghloul NA Leitch CC Lawson SS Kato M Beachy PA Beales PL DeMartino GN Fisher S Badano JL Katsanis N Disruption of the basal body compromises proteasomal function and perturbs intracellular Wnt response Nat Genet 2007 39 1350 1360 10.1038/ng.2007.12 17906624

[B112] Pretorius PR Baye LM Nishimura DY Searby CC Bugge K Yang B Mullins RF Stone EM Sheffield VC Slusarski DC Identification and functional analysis of the vision-specific BBS3 (ARL6) long isoform PLoS Genet 2010 6 e1000884 10.1371/journal.pgen.1000884 20333246 PMC2841623

[B113] Kim JC Ou YY Badano JL Esmail MA Leitch CC Fiedrich E Beales PL Archibald JM Katsanis N Rattner JB Leroux MR MKKS/BBS6, a divergent chaperonin-like protein linked to the obesity disorder Bardet–Biedl syndrome, is a novel centrosomal component required for cytokinesis J Cell Sci 2005 118 Pt 5 1007 1020 15731008 10.1242/jcs.01676

[B114] Ansley SJ Badano JL Blacque OE Hill J Hoskins BE Leitch CC Kim JC Ross AJ Eichers ER Teslovich TM Mah AK Johnsen RC Cavender JC Lewis RA Leroux MR Beales PL Katsanis N Basal body dysfunction is a likely cause of pleiotropic Bardet–Biedl syndrome Nature 2003 425 628 633 10.1038/nature02030 14520415

[B115] Lim YS Chua CE Tang BL Rabs and other small GTPases in ciliary transport Biol Cell 2011 103 209 221 10.1042/BC20100150 21488838

[B116] Liu X Bulgakov OV Darrow KN Pawlyk B Adamian M Liberman MC Li T Usherin is required for maintenance of retinal photoreceptors and normal development of cochlear hair cells Proc Natl Acad Sci U S A 2007 104 4413 4418 10.1073/pnas.0610950104 17360538 PMC1838616

[B117] Yang J Liu X Yue G Adamian M Bulgakov O Li T Rootletin, a novel coiled-coil protein, is a structural component of the ciliary rootlet J Cell Biol 2002 159 431 440 10.1083/jcb.200207153 12427867 PMC2173070

[B118] Wright RN Hong DH Perkins B RpgrORF15 connects to the Usher protein network through direct interactions with multiple whirlin isoforms Invest Ophthalmol Vis Sci 2012 53 1519 29 Print 2012 Mar 10.1167/iovs.11-8845 22323458 PMC3339914

[B119] Katsanis N Ansley SJ Badano JL Eichers ER Lewis RA Hoskins BE Scambler PJ Davidson WS Beales PL Lupski JR Triallelic inheritance in Bardet–Biedl syndrome, a Mendelian recessive disorder Science 2001 293 2256 2259 10.1126/science.1063525 11567139

[B120] Doherty D Parisi MA Finn LS Gunay-Aygun M Al-Mateen M Bates D Clericuzio C Demir H Dorschner M van Essen AJ Gahl WA Gentile M Gorden NT Hikida A Knutzen D Ozyurek H Phelps I Rosenthal P Verloes A Weigand H Chance PF Dobyns WB Glass IA Mutations in 3 genes (MKS3, CC2D2A and RPGRIP1L) cause COACH syndrome (Joubert syndrome with congenital hepatic fibrosis) J Med Genet 2010 47 8 21 10.1136/jmg.2009.067249 19574260 PMC3501959

[B121] Leitch CC Zaghloul NA Davis EE Stoetzel C Diaz-Font A Rix S Alfadhel M Lewis RA Eyaid W Banin E Dollfus H Beales PL Badano JL Katsanis N Hypomorphic mutations in syndromic encephalocele genes are associated with Bardet–Biedl syndrome Nat Genet 2008 40 443 448 10.1038/ng.97 18327255

[B122] Karska-Basta I Kubicka-Trzaska A Filemonowicz-Skoczek A Romanowska-Dixon B Kobylarz J Alstrom syndrome – a case report and literature review Klin Oczna 2008 110 188 192 18655459

[B123] Helou J Otto EA Attanasio M Allen SJ Parisi MA Glass I Utsch B Hashmi S Fazzi E Omran H O'Toole JF Sayer JA Hildebrandt F Mutation analysis of NPHP6/CEP290 in patients with Joubert syndrome and Senior-Loken syndrome Journal of Medical Genetics 2007 44 10 657 663 10.1136/jmg.2007.052027 17617513 PMC2597962

[B124] Roepman R Letteboer SJ Arts HH van Beersum SE Lu X Krieger E Ferreira PA Cremers FP Interaction of nephrocystin-4 and RPGRIP1 is disrupted by nephronophthisis or Leber congenital amaurosis-associated mutations Proc Natl Acad Sci U S A 2005 102 18520 18525 10.1073/pnas.0505774102 16339905 PMC1317916

[B125] Stone EM Cideciyan AV Aleman TS Scheetz TE Sumaroka A Ehlinger MA Schwartz SB Fishman GA Traboulsi EI Lam BL Fulton AB Mullins RF Sheffield VC Jacobson SG Variations in NPHP5 in patients with nonsyndromic leber congenital amaurosis and Senior–Loken syndrome Arch Ophthalmol 2011 129 81 87 10.1001/archophthalmol.2010.330 21220633 PMC3952880

[B126] Casteels I Demandt E Legius E Visual loss as the presenting sign of Jeune syndrome Eur J Paediatr Neurol 2000 4 243 247 10.1053/ejpn.2000.0313 11030072

[B127] den Hollander AI Roepman R Koenekoop RK Cremers FP Leber congenital amaurosis: genes, proteins and disease mechanisms Prog Retin Eye Res 2008 27 391 419 10.1016/j.preteyeres.2008.05.003 18632300

[B128] Vervoort R Lennon A Bird AC Tulloch B Axton R Miano MG Meindl A Meitinger T Ciccodicola A Wright AF Mutational hot spot within a new RPGR exon in X-linked retinitis pigmentosa Nat Genet 2000 25 462 466 10.1038/78182 10932196

[B129] Breuer DK Yashar BM Filippova E Hiriyanna S Lyons RH Mears AJ Asaye B Acar C Vervoort R Wright AF Musarella MA Wheeler P MacDonald I Iannaccone A Birch D Hoffman DR Fishman GA Heckenlively JR Jacobson SG Sieving PA Swaroop A A comprehensive mutation analysis of RP2 and RPGR in a North American cohort of families with X-linked retinitis pigmentosa Am J Hum Genet 2002 70 1545 1554 10.1086/340848 11992260 PMC379141

[B130] Jimeno D Feiner L Lillo C Teofilo K Goldstein LS Pierce EA Williams DS Analysis of kinesin-2 function in photoreceptor cells using synchronous Cre-loxP knockout of Kif3a with RHO-Cre Invest Ophthalmol Vis Sci 2006 47 5039 5046 10.1167/iovs.06-0032 17065525 PMC1904505

[B131] Marszalek JR Liu X Roberts EA Chui D Marth JD Williams DS Goldstein LS Genetic evidence for selective transport of opsin and arrestin by kinesin-II in mammalian photoreceptors Cell 2000 102 175 187 10.1016/S0092-8674(00)00023-4 10943838

[B132] Won J Shi LY Hicks W Wang J Hurd R Naggert JK Chang B Nishina PM Mouse model resources for vision research J Ophthalmol 2011 391384 Epub 2010 Oct 31 21052544 10.1155/2011/391384 PMC2968714

[B133] Liu Q Saveliev A Pierce EA The severity of retinal degeneration in Rp1h gene-targeted mice is dependent on genetic background Invest Ophthalmol Vis Sci 2009 50 1566 1574 19060274 10.1167/iovs.08-2776 PMC2701263

[B134] Liu J Huang Q Higdon J Liu W Xie T Yamashita T Cheon K Cheng C Zuo J Distinct gene expression profiles and reduced JNK signaling in retinitis pigmentosa caused by RP1 mutations Hum Mol Genet 2005 14 2945 2958 10.1093/hmg/ddi325 16126734

[B135] Gao J Cheon K Nusinowitz S Liu Q Bei D Atkins K Azimi A Daiger SP Farber DB Heckenlively JR Pierce EA Sullivan LS Zuo J Progressive photoreceptor degeneration, outer segment dysplasia, and rhodopsin mislocalization in mice with targeted disruption of the retinitis pigmentosa-1 (Rp1) gene Proc Natl Acad Sci U S A 2002 99 5698 5703 10.1073/pnas.042122399 11960024 PMC122834

[B136] Schwartz SB Aleman TS Cideciyan AV Swaroop A Jacobson SG Stone EM De novo mutation in the RP1 gene (Arg677ter) associated with retinitis pigmentosa Invest Ophthalmol Vis Sci 2003 44 3593 3597 10.1167/iovs.03-0155 12882812

[B137] Cideciyan AV Aleman TS Jacobson SG Khanna H Sumaroka A Aguirre GK Schwartz SB Windsor EA He S Chang B Stone EM Swaroop A Centrosomal-ciliary gene CEP290/NPHP6 mutations result in blindness with unexpected sparing of photoreceptors and visual brain: implications for therapy of Leber congenital amaurosis Hum Mutat 2007 28 1074 1083 10.1002/humu.20565 17554762

[B138] Louie CM Caridi G Lopes VS Brancati F Kispert A Lancaster MA Schlossman AM Otto EA Leitges M Grone HJ Lopez I Gudiseva HV O'Toole JF Vallespin E Ayyagari R Ayuso C Cremers FP den Hollander AI Koenekoop RK Dallapiccola B Ghiggeri GM Hildebrandt F Valente EM Williams DS Gleeson JG AHI1 is required for photoreceptor outer segment development and is a modifier for retinal degeneration in nephronophthisis Nat Genet 2010 42 175 180 10.1038/ng.519 20081859 PMC2884967

[B139] Lancaster MA Gopal DJ Kim J Saleem SN Silhavy JL Louie CM Thacker BE Williams Y Zaki MS Gleeson JG Defective Wnt-dependent cerebellar midline fusion in a mouse model of Joubert syndrome Nat Med 2011 17 726 731 10.1038/nm.2380 21623382 PMC3110639

[B140] Collin GB Won J Hicks WL Cook SA Nishina PM Naggert JK Meckelin is necessary for photoreceptor intraciliary transport and outer segment morphogenesis Invest Ophthalmol Vis Science 2012 53 967 974 Print 2012 Feb 10.1167/iovs.11-8766 PMC3317434 22247471

[B141] Bhowmick R Li M Sun J Baker SA Insinna C Besharse JC Photoreceptor IFT complexes containing chaperones, guanylyl cyclase 1 and rhodopsin Traffic 2009 10 648 663 10.1111/j.1600-0854.2009.00896.x 19302411 PMC2827254

[B142] Liem KF Jr He M, Ocbina PJ, Anderson KV: Mouse Kif7/Costal2 is a cilia-associated protein that regulates Sonic hedgehog signaling Proc Natl Acad Sci U S A 2009 106 13377 13382 19666503 10.1073/pnas.0906944106 PMC2726420

[B143] Chapple JP Hardcastle AJ Grayson C Spackman LA Willison KR Cheetham ME Mutations in the N-terminus of the X-linked retinitis pigmentosa protein RP2 interfere with the normal targeting of the protein to the plasma membrane Hum Mol Genet 2000 9 1919 1926 10.1093/hmg/9.13.1919 10942419

[B144] Evans RJ Schwarz N Nagel-Wolfrum K Wolfrum U Hardcastle AJ Cheetham ME The retinitis pigmentosa protein RP2 links pericentriolar vesicle transport between the Golgi and the primary cilium Hum Mol Genet 2010 19 1358 1367 10.1093/hmg/ddq012 20106869

[B145] Hurd T Zhou W Jenkins P Liu CJ Swaroop A Khanna H Martens J Hildebrandt F Margolis B The retinitis pigmentosa protein RP2 interacts with polycystin 2 and regulates cilia-mediated vertebrate development Hum Mol Genet 2010 19 4330 4344 10.1093/hmg/ddq355 20729296 PMC2957320

[B146] Won J Gifford E Smith RS Yi H Ferreira PA Hicks WL Li T Naggert JK Nishina PM RPGRIP1 is essential for normal rod photoreceptor outer segment elaboration and morphogenesis Hum Mol Genet 2009 18 4329 4339 10.1093/hmg/ddp385 19679561 PMC2766293

[B147] Pawlyk BS Bulgakov OV Liu X Xu X Adamian M Sun X Khani SC Berson EL Sandberg MA Li T Replacement gene therapy with a human RPGRIP1 sequence slows photoreceptor degeneration in a murine model of Leber congenital amaurosis Hum Gene Ther 2010 21 993 1004 10.1089/hum.2009.218 20384479 PMC2928706

[B148] Garcia-Gonzalo FR Corbit KC Sirerol-Piquer MS Ramaswami G Otto EA Noriega TR Seol AD Robinson JF Bennett CL Josifova DJ Garcia-Verdugo JM Katsanis N Hildebrandt F Reiter JF A transition zone complex regulates mammalian ciliogenesis and ciliary membrane composition Nat Genet 2011 43 776 784 10.1038/ng.891 21725307 PMC3145011

[B149] Thompson DA Khan NW Othman MI Chang B Jia L Grahek G Wu Z Hiriyanna S Nellissery J Li T Khanna H Colosi P Swaroop A Heckenlively JR Rd9 is a naturally occurring mouse model of a common form of retinitis pigmentosa caused by mutations in RPGR-ORF15 PloS One 2012 7 e35865 10.1371/journal.pone.0035865 22563472 PMC3341386

[B150] Huang WC Wright AF Roman AJ Cideciyan AV Manson FD Gewaily DY Schwartz SB Sadigh S Limberis MP Bell P Wilson JM Swaroop A Jacobson SG RPGR-associated retinal degeneration in human X-linked RP and a murine model Invest Ophthalmol Vis Sci 2012 53 5594 5608 Print 2012 Sep 10.1167/iovs.12-10070 22807293 PMC3422104

[B151] Jagger D Collin G Kelly J Towers E Nevill G Longo-Guess C Benson J Halsey K Dolan D Marshall J Naggert J Forge A Alstrom syndrome protein ALMS1 localizes to basal bodies of cochlear hair cells and regulates cilium-dependent planar cell polarity Hum Mol Genet 2011 20 466 481 10.1093/hmg/ddq493 21071598 PMC3016908

[B152] Huang-Doran I Semple RK Knockdown of the Alstrom syndrome-associated gene Alms1 in 3 T3-L1 preadipocytes impairs adipogenesis but has no effect on cell-autonomous insulin action Int J Obesity 2010 34 1554 1558 10.1038/ijo.2010.92 20514046

[B153] Arsov T Silva DG O'Bryan MK Sainsbury A Lee NJ Kennedy C Manji SS Nelms K Liu C Vinuesa CG de Kretser DM Goodnow CC Petrovsky N Fat aussie – a new Alstrom syndrome mouse showing a critical role for ALMS1 in obesity, diabetes, and spermatogenesis Mol Endocrinol 2006 20 1610 1622 16513793 10.1210/me.2005-0494

[B154] Collin GB Cyr E Bronson R Marshall JD Gifford EJ Hicks W Murray SA Zheng QY Smith RS Nishina PM Naggert JK Alms1-disrupted mice recapitulate human Alstrom syndrome Hum Mol Genet 2005 14 2323 2333 10.1093/hmg/ddi235 16000322 PMC2862911

[B155] Zhang Q Nishimura D Seo S Vogel T Morgan DA Searby C Bugge K Stone EM Rahmouni K Sheffield VC Bardet–Biedl syndrome 3 (Bbs3) knockout mouse model reveals common BBS-associated phenotypes and Bbs3 unique phenotypes Proc Natl Acad Sci U S A 2011 108 20678 20683 Epub 2011 Dec 2 10.1073/pnas.1113220108 22139371 PMC3251145

[B156] Davis RE Swiderski RE Rahmouni K Nishimura DY Mullins RF Agassandian K Philp AR Searby CC Andrews MP Thompson S Berry CJ Thedens DR Yang B Weiss RM Cassell MD Stone EM Sheffield VC A knockin mouse model of the Bardet–Biedl syndrome 1 M390R mutation has cilia defects, ventriculomegaly, retinopathy, and obesity Proc Natl Acad Sci U S A 2007 104 19422 19427 10.1073/pnas.0708571104 18032602 PMC2148305

[B157] Nishimura DY Fath M Mullins RF Searby C Andrews M Davis R Andorf JL Mykytyn K Swiderski RE Yang B Carmi R Stone EM Sheffield VC Bbs2-null mice have neurosensory deficits, a defect in social dominance, and retinopathy associated with mislocalization of rhodopsin Proc Natl Acad Sci U S A 2004 101 16588 16593 10.1073/pnas.0405496101 15539463 PMC534519

[B158] Rahmouni K Fath MA Seo S Thedens DR Berry CJ Weiss R Nishimura DY Sheffield VC Leptin resistance contributes to obesity and hypertension in mouse models of Bardet–Biedl syndrome J Clin Invest 2008 118 1458 1467 10.1172/JCI32357 18317593 PMC2262028

[B159] Abd-El-Barr MM Sykoudis K Andrabi S Eichers ER Pennesi ME Tan PL Wilson JH Katsanis N Lupski JR Wu SM Impaired photoreceptor protein transport and synaptic transmission in a mouse model of Bardet–Biedl syndrome Vis Res 2007 47 3394 3407 10.1016/j.visres.2007.09.016 18022666 PMC2661240

[B160] Mykytyn K Mullins RF Andrews M Chiang AP Swiderski RE Yang B Braun T Casavant T Stone EM Sheffield VC Bardet–Biedl syndrome type 4 (BBS4)-null mice implicate Bbs4 in flagella formation but not global cilia assembly Proc Natl Acad Sci U S A 2004 101 8664 8669 10.1073/pnas.0402354101 15173597 PMC423252

[B161] Simons DL Boye SL Hauswirth WW Wu SM Gene therapy prevents photoreceptor death and preserves retinal function in a Bardet–Biedl syndrome mouse model Proc Natl Acad Sci U S A 2011 108 6276 6281 10.1073/pnas.1019222108 21444805 PMC3076852

[B162] Tadenev AL Kulaga HM May-Simera HL Kelley MW Katsanis N Reed RR Loss of Bardet–Biedl syndrome protein-8 (BBS8) perturbs olfactory function, protein localization, and axon targeting Proc Natl Acad Sci U S A 2011 108 10320 10325 10.1073/pnas.1016531108 21646512 PMC3121838

[B163] Fath MA Mullins RF Searby C Nishimura DY Wei J Rahmouni K Davis RE Tayeh MK Andrews M Yang B Sigmund CD Stone EM Sheffield VC Mkks-null mice have a phenotype resembling Bardet–Biedl syndrome Hum Mol Genet 2005 14 1109 1118 10.1093/hmg/ddi123 15772095

[B164] Sato T Mushiake S Kato Y Sato K Sato M Takeda N Ozono K Miki K Kubo Y Tsuji A Harada R Harada A The Rab8 GTPase regulates apical protein localization in intestinal cells Nature 2007 448 366 369 10.1038/nature05929 17597763

[B165] Kudryashova E Wu J Havton LA Spencer MJ Deficiency of the E3 ubiquitin ligase TRIM32 in mice leads to a myopathy with a neurogenic component Hum Mol Genet 2009 18 1353 1367 10.1093/hmg/ddp036 19155210 PMC2722196

[B166] Brancati F Barrano G Silhavy JL Marsh SE Travaglini L Bielas SL Amorini M Zablocka D Kayserili H Al-Gazali L Bertini E Boltshauser E D'Hooghe M Fazzi E Fenerci EY Hennekam RC Kiss A Lees MM Marco E Phadke SR Rigoli L Romano S Salpietro CD Sherr EH Signorini S Stromme P Stuart B Sztriha L Viskochil DH Yuksel A CEP290 mutations are frequently identified in the oculo-renal form of Joubert syndrome-related disorders Am J Hum Genet 2007 81 104 113 10.1086/519026 17564967 PMC1950920

[B167] Valente EM Silhavy JL Brancati F Barrano G Krishnaswami SR Castori M Lancaster MA Boltshauser E Boccone L Al-Gazali L Fazzi E Signorini S Louie CM Bellacchio E Bertini E Dallapiccola B Gleeson JG Mutations in CEP290, which encodes a centrosomal protein, cause pleiotropic forms of Joubert syndrome Nat Genet 2006 38 623 625 10.1038/ng1805 16682970

[B168] Coppieters F Lefever S Leroy BP De Baere E CEP290, a gene with many faces: mutation overview and presentation of CEP290base Hum Mutat 2010 31 1097 1108 10.1002/humu.21337 20690115

[B169] Coppieters F Casteels I Meire F De Jaegere S Hooghe S van Regemorter N Van Esch H Matuleviciene A Nunes L Meersschaut V Walraedt S Standaert L Coucke P Hoeben H Kroes HY Vande Walle J de Ravel T Leroy BP De Baere E Genetic screening of LCA in Belgium: predominance of CEP290 and identification of potential modifier alleles in AHI1 of CEP290-related phenotypes Hum Mutat 2010 31 E1709 E1766 10.1002/humu.21336 20683928 PMC3048164

[B170] Morecroft I Doyle B Nilsen M Kolch W Mair K Maclean MR Mice lacking the Raf-1 kinase inhibitor protein exhibit exaggerated hypoxia-induced pulmonary hypertension Br J Pharmacol 2011 163 948 963 10.1111/j.1476-5381.2011.01305.x 21385176 PMC3130942

[B171] Iannaccone A Wang X Jablonski MM Kuo SF Baldi A Cosgrove D Morton CC Swaroop A Increasing evidence for syndromic phenotypes associated with RPGR mutations Am J Ophthalmol 2004 137 785 786 author reply 786 15059739 10.1016/j.ajo.2003.11.050

[B172] Shu X Black GC Rice JM Hart-Holden N Jones A O'Grady A Ramsden S Wright AF RPGR mutation analysis and disease: an update Hum Mutat 2007 28 322 328 10.1002/humu.20461 17195164

[B173] Zito I Downes SM Patel RJ Cheetham ME Ebenezer ND Jenkins SA Bhattacharya SS Webster AR Holder GE Bird AC Bamiou DE Hardcastle AJ RPGR mutation associated with retinitis pigmentosa, impaired hearing, and sinorespiratory infections J Med Genet 2003 40 609 615 10.1136/jmg.40.8.609 12920075 PMC1735548

[B174] Schmid F Glaus E Cremers FP Kloeckener-Gruissem B Berger W Neidhardt J Mutation- and tissue-specific alterations of RPGR transcripts Invest Ophthalmol Vis Sci 2010 51 1628 1635 10.1167/iovs.09-4031 19834030

[B175] Hong DH Li T Complex expression pattern of RPGR reveals a role for purine-rich exonic splicing enhancers Invest Ophthalmol Vis Sci 2002 43 3373 3382 12407146

[B176] He S Parapuram SK Hurd TW Behnam B Margolis B Swaroop A Khanna H Retinitis Pigmentosa GTPase Regulator (RPGR) protein isoforms in mammalian retina: insights into X-linked Retinitis Pigmentosa and associated ciliopathies Vision Res 2008 48 366 376 10.1016/j.visres.2007.08.005 17904189 PMC2267686

[B177] Meindl A Dry K Herrmann K Manson F Ciccodicola A Edgar A Carvalho MR Achatz H Hellebrand H Lennon A Migliaccio C Porter K Zrenner E Bird A Jay M Lorenz B Wittwer B D'Urso M Meitinger T Wright A A gene (RPGR) with homology to the RCC1 guanine nucleotide exchange factor is mutated in X-linked retinitis pigmentosa (RP3) Nat Genet 1996 13 35 42 10.1038/ng0596-35 8673101

[B178] Yan D Swain PK Breuer D Tucker RM Wu W Fujita R Rehemtulla A Burke D Swaroop A Biochemical characterization and subcellular localization of the mouse retinitis pigmentosa GTPase regulator (mRpgr) J Biol Chem 1998 273 19656 19663 10.1074/jbc.273.31.19656 9677393

[B179] Iannaccone A Breuer DK Wang XF Kuo SF Normando EM Filippova E Baldi A Hiriyanna S MacDonald CB Baldi F Cosgrove D Morton CC Swaroop A Jablonski MM Clinical and immunohistochemical evidence for an X linked retinitis pigmentosa syndrome with recurrent infections and hearing loss in association with an RPGR mutation J Med Genet 2003 40 e118 10.1136/jmg.40.11.e118 14627685 PMC1735323

[B180] Sharon D Sandberg MA Rabe VW Stillberger M Dryja TP Berson EL RP2 and RPGR mutations and clinical correlations in patients with X-linked retinitis pigmentosa Am J Hum Genet 2003 73 1131 1146 10.1086/379379 14564670 PMC1180492

[B181] Ocbina PJ Eggenschwiler JT Moskowitz I Anderson KV Complex interactions between genes controlling trafficking in primary cilia Nat Genet 2011 43 547 553 10.1038/ng.832 21552265 PMC3132150

[B182] Friedman TB Schultz JM Ahmed ZM Tsilou ET Brewer CC Usher syndrome: hearing loss with vision loss Adv Otorhinolaryngol 2011 70 56 65 21358186 10.1159/000322473

[B183] Murga-Zamalloa CA Swaroop A Khanna H RPGR-containing protein complexes in syndromic and non-syndromic retinal degeneration due to ciliary dysfunction J Genet 2009 88 399 407 10.1007/s12041-009-0061-7 20090203 PMC3464916

[B184] Travaglini L Brancati F Attie-Bitach T Audollent S Bertini E Kaplan J Perrault I Iannicelli M Mancuso B Rigoli L Rozet JM Swistun D Tolentino J Dallapiccola B Gleeson JG Valente EM Zankl A Leventer R Grattan-Smith P Janecke A D'Hooghe M Sznajer Y Van Coster R Demerleir L Dias K Moco C Moreira A Kim CA Maegawa G Petkovic D Expanding CEP290 mutational spectrum in ciliopathies Am J Med Genet A 2009 149A 2173 2180 10.1002/ajmg.a.33025 19764032 PMC4340070

[B185] Chaki M Hoefele J Allen SJ Ramaswami G Janssen S Bergmann C Heckenlively JR Otto EA Hildebrandt F Genotype–phenotype correlation in 440 patients with NPHP-related ciliopathies Kidney Int 2011 80 1239 1245 10.1038/ki.2011.284 21866095 PMC4037742

[B186] Zhang Q Seo S Bugge K Stone EM Sheffield VC BBS proteins interact genetically with the IFT pathway to influence SHH related phenotypes Hum Mol Genet 2012 21 1945 1953 Epub 2012 Jan 6 10.1093/hmg/dds004 22228099 PMC3315203

[B187] Badano JL Kim JC Hoskins BE Lewis RA Ansley SJ Cutler DJ Castellan C Beales PL Leroux MR Katsanis N Heterozygous mutations in BBS1, BBS2 and BBS6 have a potential epistatic effect on Bardet–Biedl patients with two mutations at a second BBS locus Hum Mol Genet 2003 12 1651 1659 10.1093/hmg/ddg188 12837689

[B188] Beales PL Badano JL Ross AJ Ansley SJ Hoskins BE Kirsten B Mein CA Froguel P Scambler PJ Lewis RA Lupski JR Katsanis N Genetic interaction of BBS1 mutations with alleles at other BBS loci can result in non-Mendelian Bardet–Biedl syndrome Am J Hum Genet 2003 72 1187 1199 10.1086/375178 12677556 PMC1180271

[B189] Bin J Madhavan J Ferrini W Mok CA Billingsley G Heon E BBS7 and TTC8 (BBS8) mutations play a minor role in the mutational load of Bardet–Biedl syndrome in a multiethnic population Hum Mutat 2009 30 E737 E746 10.1002/humu.21040 19402160

[B190] Katsanis N Eichers ER Ansley SJ Lewis RA Kayserili H Hoskins BE Scambler PJ Beales PL Lupski JR BBS4 is a minor contributor to Bardet–Biedl syndrome and may also participate in triallelic inheritance Am J Hum Genet 2002 71 22 29 10.1086/341031 12016587 PMC384990

[B191] Takeda S Narita K Structure and function of vertebrate cilia, towards a new taxonomy Differentiation 2011 83 S4 S11 Epub 2011 Nov 25 22118931 10.1016/j.diff.2011.11.002

[B192] Nachury MV Loktev AV Zhang Q Westlake CJ Peranen J Merdes A Slusarski DC Scheller RH Bazan JF Sheffield VC Jackson PK A core complex of BBS proteins cooperates with the GTPase Rab8 to promote ciliary membrane biogenesis Cell 2007 129 1201 1213 10.1016/j.cell.2007.03.053 17574030

[B193] Sang L Miller JJ Corbit KC Giles RH Brauer MJ Otto EA Baye LM Wen X Scales SJ Kwong M Huntzicker EG Sfakianos MK Sandoval W Bazan JF Kulkarni P Garcia-Gonzalo FR Seol AD O'Toole JF Held S Reutter HM Lane WS Rafiq MA Noor A Ansar M Devi AR Sheffield VC Slusarski DC Vincent JB Doherty DA Hildebrandt F Mapping the NPHP–JBTS–MKS protein network reveals ciliopathy disease genes and pathways Cell 2011 145 513 528 10.1016/j.cell.2011.04.019 21565611 PMC3383065

[B194] van Reeuwijk J Arts HH Roepman R Scrutinizing ciliopathies by unraveling ciliary interaction networks Hum Mol Genet 2011 20 R2 R149 R157 10.1093/hmg/ddr354 21862450

